# Gasdermin B-mediated pyroptosis as a host defense against swine enteric coronaviruses and its antagonism by PEDV

**DOI:** 10.1128/mbio.02904-25

**Published:** 2025-12-18

**Authors:** Yao Yao, Ning Huang, Xinyu Huang, Mengqi Yuan, Li Kang, Yanlong Ma, Jun Han, Guozhong Zhang, Pinghuang Liu

**Affiliations:** 1 State Key Laboratory of Veterinary Public Health and Safety, College of Veterinary Medicine, China Agricultural University 630101 , Beijing, China; 2 Key Laboratory of Animal Epidemiology of the Ministry of Agriculture and Rural Affairs, College of Veterinary Medicine, China Agricultural University 630101 , Beijing, China; Huazhong Agricultural University, Wuhan, Hubei, China

**Keywords:** gasdermin B, pyroptosis, coronaviruses, porcine epidemic diarrhea virus (PEDV), caspases

## Abstract

Gasdermin B (GSDMB), a member of the spore-forming protein gasdermin (GSDM) family, is critical for inflammation and immunity and has been genetically linked to human diseases. Despite its prominent expression at mucosal surfaces, including the gastrointestinal and respiratory tracts, GSDMB’s role in defending against viral pathogens at these barrier tissues remains poorly defined. Here, we reveal that porcine GSDMB (pGSDMB), which is highly expressed in the intestinal epithelium, is a potent innate restriction factor against porcine epidemic diarrhea virus (PEDV), a major enteric coronavirus. Mechanistically, PEDV infection activated caspase-3/6/7 to cleave pGSDMB at D237, generating an active N-terminal fragment (pGSDMB_1–237_) that triggered pyroptotic cell death to limit viral propagation. Conversely, PEDV evolved a sophisticated countermeasure: the viral nonstructural proteins nsp1 and nsp15 cooperatively suppressed pGSDMB protein expression. This immune evasion required a critical region within nsp1 (86–110 amino acids) and the catalytic endoribonuclease residues (H226 and H241) of nsp15. Importantly, pGSDMB-mediated pyroptosis broadly inhibited replication of diverse swine enteric coronaviruses, including transmissible gastroenteritis virus and porcine deltacoronavirus. Our findings establish GSDMB as an executor of pyroptosis that guards the mucosal interface against coronavirus infection and unveils a novel viral strategy to circumvent this defense, highlighting new avenues for therapeutic intervention against coronaviruses.

**IMPORTANCE:**

While gasdermin B (GSDMB) is genetically associated with mucosal inflammatory diseases like asthma, its function in host defense at mucosal barriers remains an open question. This study defines a critical role for GSDMB as a central innate immune executor against enteric coronaviruses. We demonstrate that porcine GSDMB (pGSDMB) is cleaved during infection to trigger pyroptotic cell death, thereby restricting the replication of porcine epidemic diarrhea virus (PEDV) and other swine enteric coronaviruses. Furthermore, we identify a novel immune evasion strategy whereby PEDV employs its nsp1 and nsp15 proteins to suppress pGSDMB expression, delineating the key viral domains required for this countermeasure. These findings bridge a significant knowledge gap by revealing GSDMB as a guardian of the mucosal interface and inform the development of potential broad-acting therapeutic strategies against coronaviruses.

## INTRODUCTION

Gasdermins (GSDMs) have attracted considerable scientific interest due to their critical roles in innate immunity, host defense, inflammation, cancer, and other diseases. In the human genome, the GSDMs comprise six members: GSDMA, GSDMB, GSDMC, GSDMD, GSDME (DFNA5), and DFNB59 (PVJK) (1). Except for DFNB59, GSDMs consist of an N-terminal pore-forming domain (NTD) and a C-terminal autoinhibitory domain (CTD), which maintain autoinhibition through intramolecular interactions in the resting state (2). Among them, GSDMB is the most recently evolved member of the GSDM family, widely present in many mammalian species but absent in mice and rats (3). Human GSDMB (hGSDMB), located on chromosome 17q21.1, comprises 11 exons and gives rise to multiple isoforms through alternative promoters and splicing, with five transcript variants (isoforms 1–5) listed in the National Center for Biotechnology Information (NCBI) database (4). In humans, GSDMB shows broad tissue distribution, with its expression most pronounced in the epithelial cells of the respiratory and digestive tracts (5). Multiple genome-wide association studies have identified several single-nucleotide polymorphisms within or near the *GSDMB* gene that are strongly associated with asthma, and elevated GSDMB expression has been shown to correlate with increased disease severity and frequency of exacerbations (6–9). However, the role of GSDMB in the host defense at mucosal barriers, particularly in enteric virus infection, remains incompletely understood.

Swine enteric coronaviruses (SECoVs) are major pathogens causing acute watery diarrhea in neonatal piglets, primarily including porcine epidemic diarrhea virus (PEDV), transmissible gastroenteritis virus (TGEV), and porcine deltacoronavirus (PDCoV) (10). Among them, PEDV poses the greatest threat to neonatal piglets due to its high mortality and rapid spread, making it a primary focus of current research (11, 12). PEDV is an enveloped, positive-sense single-stranded RNA virus belonging to the genus *Alphacoronavirus* in the family *Coronaviridae* (13). The viral genome is approximately 28 kb in length and contains more than seven open reading frames (ORFs), which encode four structural proteins—spike, envelope, membrane, and nucleocapsid—16 nonstructural proteins (nsp1–nsp16), and a single accessory protein, ORF3 (14). PEDV primarily infects the small intestine *in vivo*, causing cell swelling and fusion, which leads to extensive death and shedding of enterocytes and goblet cells, accompanied by a dysfunction in nutrition intake and serious diarrhea (15, 16). Previous study has demonstrated that diarrhea can function as a mechanical host defense to expel pathogens such as rotavirus (17). However, the specific interplay between PEDV and the intestinal barrier, particularly the role of cell death pathways in both viral defense and pathogenesis, remains poorly defined.

Pyroptosis is a lytic and inflammatory type of programmed cell death mediated by the GSDMs, characterized by membrane pore formation, cell swelling, and release of intracellular contents, playing a critical regulatory role in host defense and inflammatory responses (18). In resting cells, GSDMs reside in the cytosol in an autoinhibited form. Upon stimulation by danger signals, they are specifically cleaved by cytosolic proteases such as caspases and granzymes, releasing the NTD, which translocates to the plasma membrane to form pores, thereby inducing pyroptotic cell death and interleukin-1β release (19). Among them, GSDMD is the first to be identified as possessing pore-forming activity that mediates pyroptosis, whereas the pore-forming function of GSDMB has long been a subject of debate. Not until 2023 did several studies demonstrate that only the released NTD of isoforms 3 and 4, which contain exon 6, exhibit pore-forming activity in liposomes, Gram-negative bacterial membranes, and cancer cell lines (20, 21). Additionally, the specific caspases responsible for mediating GSDM cleavage remain controversial, particularly for GSDMB. As key executioners of pyroptosis, GSDMs play an important defensive role in host resistance to pathogen invasion. In our previous study, we have revealed that porcine GSDMD (pGSDMD), through its N-terminal pore-forming activity, facilitates the release of interferon (IFN)-β, which in turn effectively suppresses TGEV and PDCoV infections (22). However, while prominent expression at mucosal surfaces, including the gastrointestinal and respiratory tracts, the functional roles and mechanisms of GSDMB in SECoVs infection remain poorly understood.

To achieve efficient replication and persistent infection within host cells, viruses have evolved multiple strategies to evade host antiviral immune responses, among which the interference of host antiviral protein expression by viral proteins is a key mechanism. In coronaviruses, nsp1 is unique to α-coronaviruses and β-coronaviruses and evades host antiviral immune responses by inhibiting the synthesis of host antiviral proteins through multiple strategies, including blocking mRNA nuclear export, cleaving host mRNAs, and obstructing translation initiation (23). For example, severe acute respiratory syndrome coronavirus 2 nsp1 binds to the 18S rRNA within the ribosomal mRNA entry channel, downregulating IFN-β protein expression and thereby suppressing the innate immune response to viral detection (24). Notably, unique sequences within the 5′ untranslated region (5′UTR) of coronavirus mRNAs enable viral protein synthesis to evade nsp1-mediated inhibition (25). Besides nsp1, the coronavirus endoribonuclease (EndoU) nsp15 similarly functions to inhibit host protein synthesis. A recent study has reported that coronavirus nsp15 suppresses host protein synthesis by targeting host factors involved in the translation machinery, including host RNA and PABPC1, and this inhibitory effect depends on its EndoU activity (26). However, reports on how coronaviruses exploit their own proteins to modulate GSDMB-mediated immune responses remain scarce.

In this study, we demonstrated that pGSDMB, which is highly expressed in the porcine intestine, markedly suppresses PEDV replication. Mechanistically, PEDV infection induced caspase-3/6/7-mediated cleavage of pGSDMB at residue D237, generating the functional fragment GSDMB_1–237_ that triggers pyroptosis. Interestingly, PEDV counteracted this antiviral effect by suppressing pGSDMB protein expression via nsp1 and nsp15, which rely on the critical domain spanning residues 86–110 in nsp1 and the EndoU activity of nsp15. Notably, pGSDMB also exhibited broad-spectrum antiviral activity by restricting replication of other SECoVs, including TGEV and PDCoV, through pyroptosis. Collectively, our findings unveil GSDMB as a critical mediator of antiviral pyroptosis against coronaviruses and delineate the mechanism by which PEDV evades this innate immune defense, providing a foundational framework for developing novel therapeutics.

## RESULTS

### pGSDMB significantly inhibits PEDV replication

**Fig 1:**
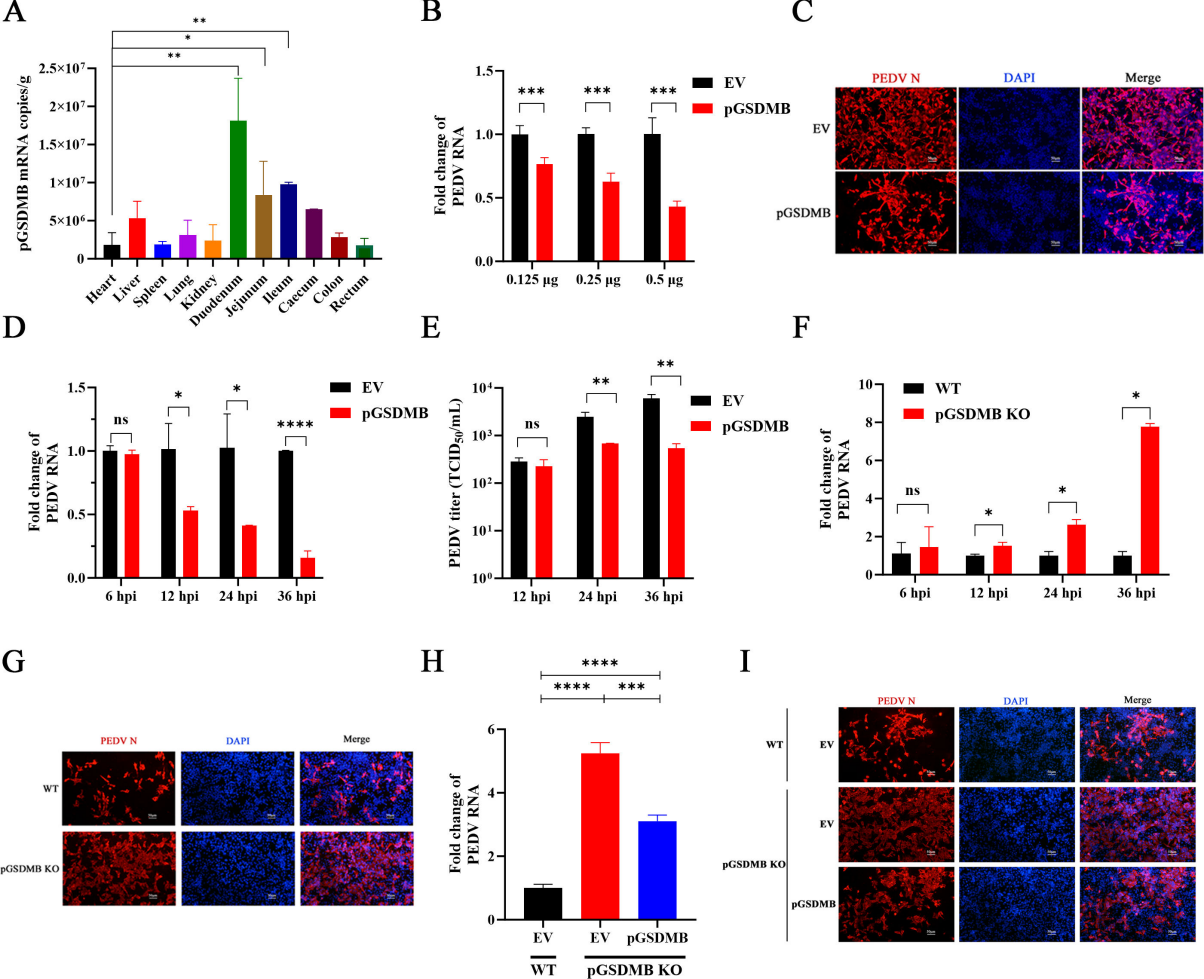
pGSDMB significantly inhibits PEDV replication. (**A**) Tissue distribution of pGSDMB mRNA in pigs determined by RT-qPCR. Approximately 0.3 g of each tissue was homogenized, and equal volumes of RNA were extracted and reverse-transcribed into cDNA. A standard curve of pGSDMB was used to calculate its absolute copy number in each tissue. (**B–C**) IPI-2I cells were transfected with empty vector (EV) or different concentrations of pGSDMB for 24 h prior to PEDV infection (MOI = 0.1). Viral genome expression was measured by RT-qPCR (**B**), and PEDV N protein was detected by IFA (**C**) at 24 hpi. (**D–E**) IPI-2I cells were transfected with pGSDMB for 24 h and then infected with PEDV (MOI = 0.1) for the indicated times. Viral genome expression was quantified by RT-qPCR (**D**), and viral titers were determined by TCID₅₀ assay (**E**). (**F–G**) Wild-type (WT) or pGSDMB-KO IPI-2I cells were infected with PEDV (MOI = 0.1). Viral genome expression at the indicated time points was quantified by RT-qPCR (**F**), and PEDV N protein was detected by IFA at 24 hpi (**G**). (**H–I**) Wild-type or pGSDMB-KO cells were transfected with empty vector or pGSDMB, followed by PEDV infection (MOI = 0.1). At 24 hpi, cells were harvested to assess viral replication by RT-qPCR (**H**) or IFA (**I**). Results are expressed as means ± standard deviation from three independent experiments. *P* values were analyzed using Student’s *t-*test. *P < 0.05; **P < 0.01; ***P < 0.001; ****P < 0.0001; ns, not significant.

In humans, GDSMB is widely expressed across various tissues and organs, with particularly high levels in the gastrointestinal tract (27). To investigate the distribution of pGSDMB *in vivo*, we assessed its expression levels in various porcine tissues. Consistent with this, pGSDMB exhibited high expression in digestive tract tissues, especially the small intestine, and the lowest expression in the heart (Fig. 1A), suggesting potential important roles for pGSDMB in the physiological and anti-infection functions of the porcine digestive system. To investigate the effect of pGSDMB on porcine intestinal coronavirus PEDV infection, we transfected pGSDMB into porcine intestinal epithelial IPI-2I cells and infected them with PEDV. Quantitative real-time PCR (RT-qPCR) analysis revealed that pGSDMB inhibited PEDV infection in a dose-dependent manner, which was further confirmed by immunofluorescence assay (IFA) targeting the PEDV N protein (Fig. 1B and C). The growth kinetics of PEDV in IPI-2I cells revealed that viral genome levels in pGSDMB-overexpressing cells were markedly reduced from 12-h post-infection (hpi) to 36 hpi, indicating that pGSDMB exerts its inhibitory effect during the viral replication phase (Fig. 1D). Consistently, the viral titers in the supernatants of pGSDMB-overexpressing cells were significantly lower than those in the control group (Fig. 1E). To further investigate the effect of pGSDMB on PEDV replication, we generated a stable pGSDMB-knockout (pGSDMB-KO) IPI-2I cell line using CRISPR/Cas9 technology, with knockout efficiency confirmed by Sanger sequencing and no detectable effects on cell viability or proliferation under normal conditions (Fig. S1A through C). Compared with wild-type cells, pGSDMB-KO cells exhibited significantly higher levels of PEDV genomic RNA and N protein expressions, which was the opposite of the effect observed upon pGSDMB overexpression (Fig. 1F and G). Furthermore, reintroduction of pGSDMB into pGSDMB-KO cells mitigated the enhanced effect of PEDV infection in pGSDMB-KO cells, suggesting the inhibitory role of pGSDMB in PEDV replication (Fig. 1H and I). Overall, these data suggest that pGSDMB, highly expressed in the small intestine, suppresses PEDV replication during the late stage of infection.

### pGSDMB inhibits PEDV replication independently of IFN responses

**Fig 2:**
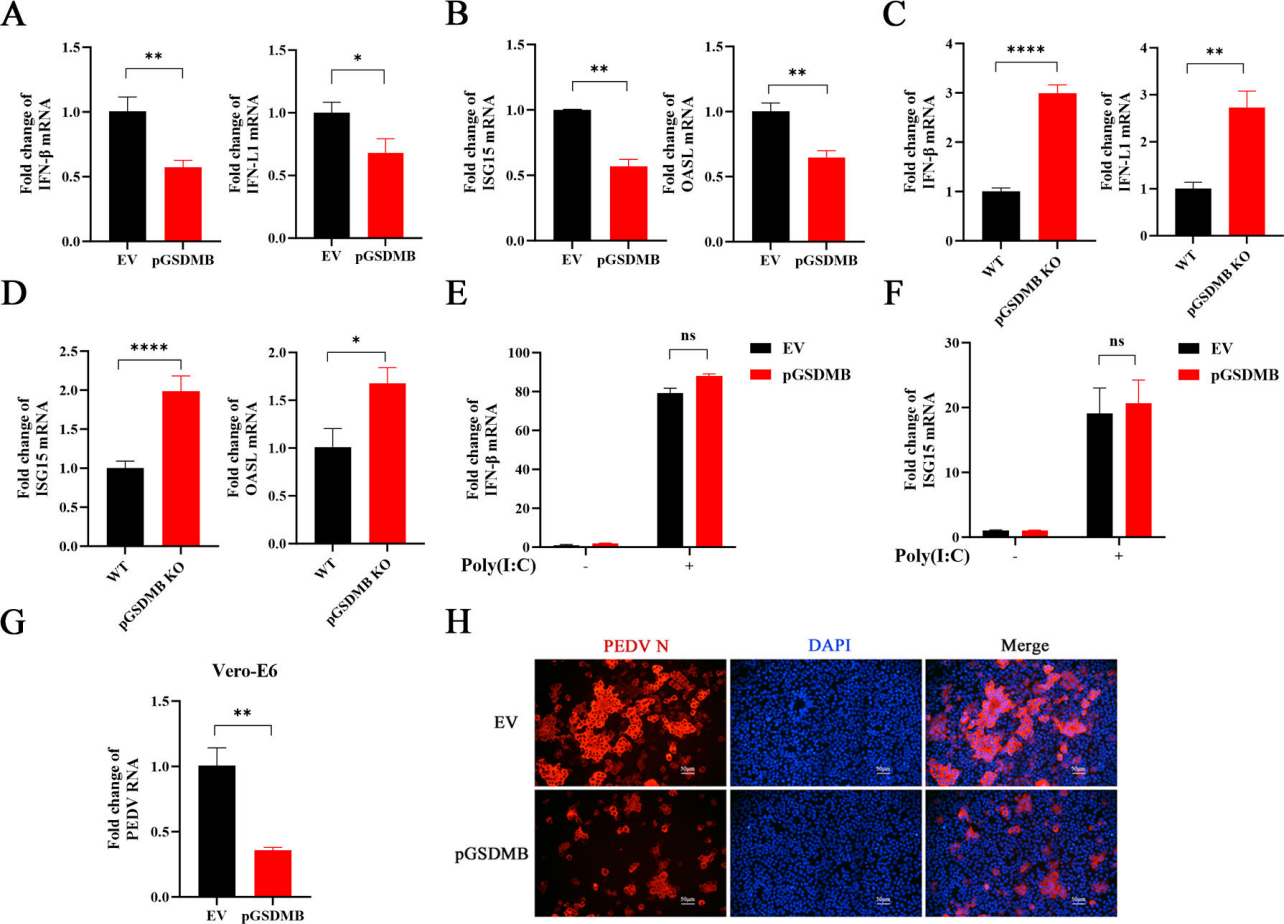
pGSDMB inhibits PEDV replication independently of IFN responses. (**A–B**) IPI-2I cells were transfected with empty vector or pGSDMB for 24 h prior to PEDV infection. The mRNA expression levels of IFNs (IFN-β and IFN-λ1) (**A**) and ISGs (ISG15 and OASL) (**B**) were measured by RT-qPCR at 24 hpi. (**C–D**) Wild-type or pGSDMB-KO IPI-2I cells were infected with PEDV for 24 h. The mRNA expression levels of IFNs (IFN-β and IFN-λ1) (**C**) and ISGs (ISG15 and OASL) (**D**) were measured by RT-qPCR. (**E–F**) IPI-2I cells were transfected with empty vector or pGSDMB and then stimulated with 1 µg/mL poly(I:C) for 12 h. The mRNA expression levels of IFN-β (**E**) and ISG15 (**F**) were subsequently measured by RT-qPCR. (**G–H**) Vero-E6 cells were transfected with empty vector or pGSDMB for 24 h and infected with PEDV. Viral replication was assessed at 24 hpi by RT-qPCR (**G**) or IFA (**H**), respectively. Results are expressed as means ± standard deviation from three independent experiments. *P* values were analyzed using Student’s *t-*test. *P < 0.05; **P < 0.01; ***P < 0.001; ****P < 0.0001; ns, not significant.

IFNs and IFN-stimulated genes (ISGs) play pivotal roles in controlling viral infections. In our previous study, pGSDMD was found to promote IFN-β release through its N-terminal pore-forming activity, thereby enhancing the expression of ISGs and effectively inhibiting TGEV and PDCoV infections (22). Therefore, we sought to investigate whether pGSDMB exerts its antiviral effect against PEDV by regulating IFN responses. Interestingly, overexpression of pGSDMB markedly suppressed PEDV infection-induced expression of IFN-β, IFN-λ1, ISG15, and OASL (Fig. 2A and B). In contrast, pGSDMB-KO significantly enhanced the PEDV-elicited expression of these IFNs and ISGs (Fig. 2C and D). To further validate the role of pGSDMB in modulating IFNs and ISGs expression, we next examined the effect of pGSDMB on IFN-β and ISG15 expression induced by poly(I:C), a structural analog of double-stranded RNA that can robustly induce type I IFN production. As shown in Fig. 2E and F, poly(I:C) stimulation induced comparable levels of IFN-β and ISG15 expression in both control and pGSDMB-overexpressing cells. These demonstrate that pGSDMB does not directly inhibit virus-induced IFN responses. To further validate, we evaluated the effect of pGSDMB on PEDV infection in African green monkey kidney (Vero-E6) cells, which are genetically deficient in producing type I IFNs (28). As expected, the ability of pGSDMB to suppress PEDV replication was not diminished in Vero-E6 cells (Fig. 2G), which was consistent with the observations from IFA (Fig. 2H). Together, these results indicate that pGSDMB inhibits PEDV replication independently of the IFN responses.

### pGSDMB plays a critical role in pyroptosis induced by PEDV infection

**Fig 3:**
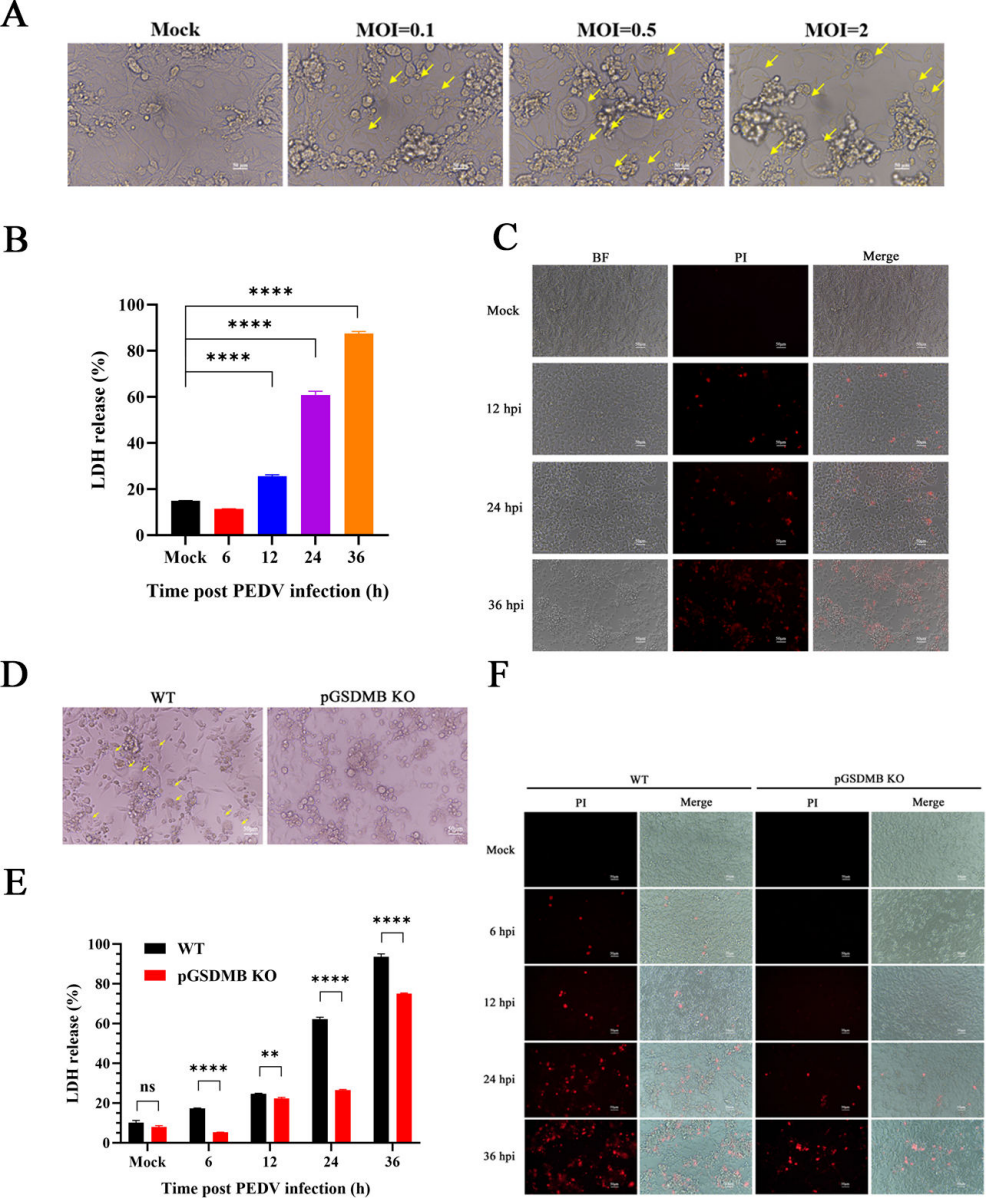
pGSDMB plays a critical role in pyroptosis induced by PEDV infection. (**A**) IPI-2I cells were infected with PEDV at different MOIs (0.1, 0.5, and 2). At 24 hpi, pyroptotic morphological changes were observed under a light microscope, with yellow arrows highlighting pyroptotic cells. (**B–C**) Following PEDV infection of IPI-2I cells, pyroptosis was assessed at different time points by measuring LDH release in the supernatant (**B**) or by PI staining (**C**), with red fluorescence indicating PI^+^ cells. “BF” denotes bright field. (**D**) Wild-type or pGSDMB-KO IPI-2I cells were infected with PEDV. At 24 hpi, pyroptotic morphological changes were examined under a light microscope, with yellow arrows indicating pyroptotic cells. (**E–F**) Following PEDV infection of wild-type or pGSDMB-KO IPI-2I cells, pyroptosis was assessed at different time points by measuring LDH release in the supernatant (**E**) or by PI staining (**F**), with red fluorescence indicating PI^+^ cells. Results are expressed as means ± standard deviation from three independent experiments. *P* values were analyzed using Student’s *t-*test. *P < 0.05; **P < 0.01; ***P < 0.001; ****P < 0.0001; ns, not significant.

Pyroptosis is a form of lytic, programmed cell death and can function as an innate host defense against pathogenic microorganisms including viruses (18, 29). As a member of the GSDMs, GSDMB also possesses the ability to induce pyroptosis (30). Therefore, we hypothesized that pGSDMB may inhibit PEDV replication by triggering pyroptosis. First, we investigated whether PEDV infection could induce pyroptosis by infecting IPI-2I cells with PEDV at different multiplicities of infection (MOIs). As shown in Fig. 3A, PEDV-infected cells exhibited characteristic morphological changes consistent with pyroptosis, including cell rounding, swelling, and a “fried-egg” appearance due to the protruding nucleus. Consistent with these morphological changes, PEDV infection markedly increased the proportion of propidium iodide-positive (PI^+^) cells and the release of lactate dehydrogenase (LDH) in IPI-2I cells, with both effects becoming more pronounced over time (Fig. 3B and C). To clarify the role of pGSDMB in PEDV-induced pyroptosis, we infected pGSDMB-KO cells with PEDV. Compared with wild-type IPI-2I cells, the number of cells showing rounding and swelling was significantly decreased in pGSDMB-KO cells under PEDV infection (Fig. 3D). Consistently, both the proportion of PI^+^ cells and the release of LDH were markedly reduced (Fig. 3E and F), further supporting the critical role of pGSDMB in PEDV-induced pyroptosis. These data indicate that PEDV infection induces pyroptosis, and pGSDMB plays a key role in this process.

### pGSDMB_1–237_ inhibits PEDV replication by inducing pyroptosis

**Fig 4:**
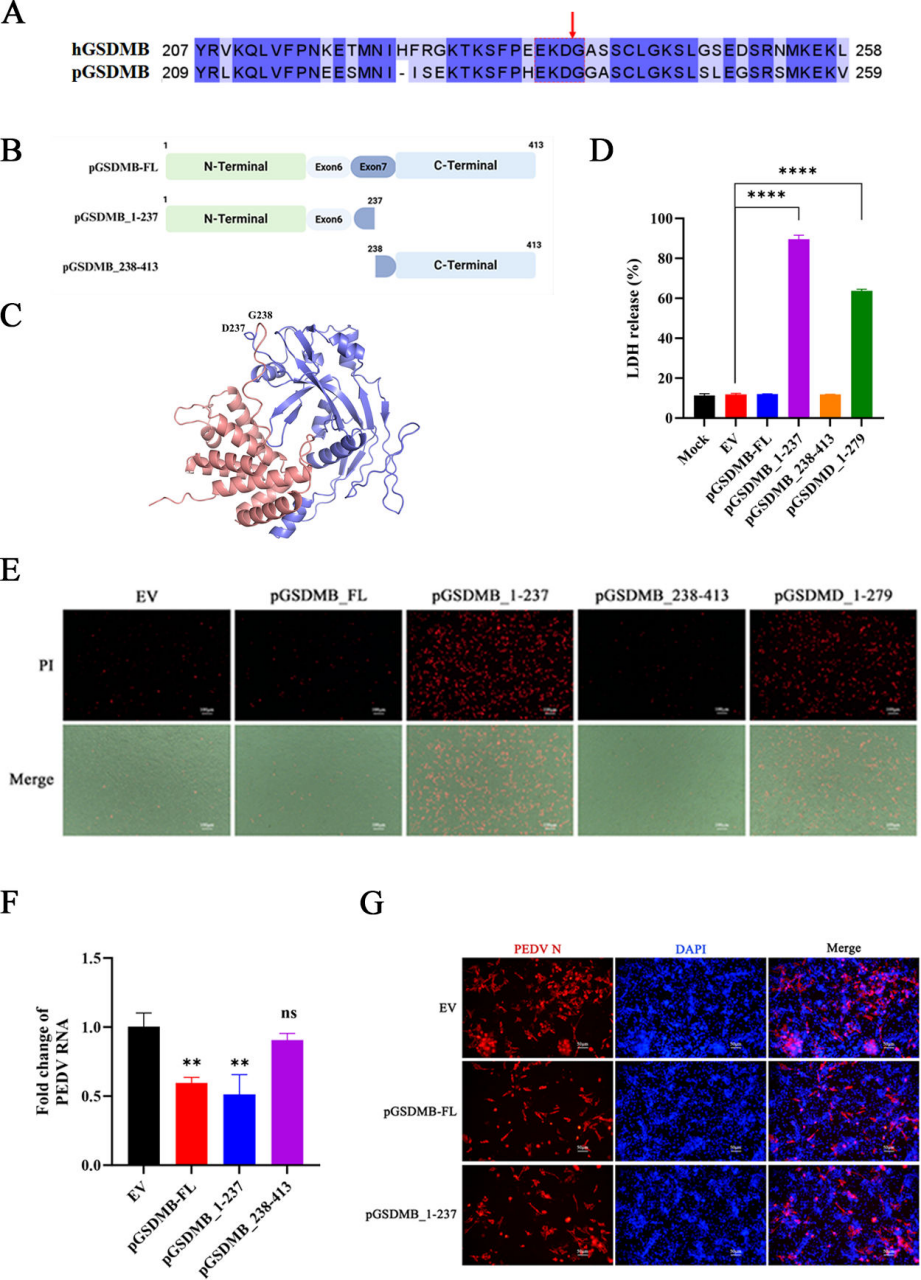
pGSDMB_1–237_ inhibits PEDV replication by inducing pyroptosis. (**A**) Amino acid sequence comparison between hGSDMB and pGSDMB, with red arrows indicating the predicted cleavage sites. (**B**) Schematic representation of pGSDMB and its truncated mutants, showing the NTD (1–237 aa) and the CTD (238–413 aa). (**C**) Predicted three-dimensional structure of pGSDMB generated by AlphaFold3, with the NTD in blue and the CTD in red. (**D–E**) pGSDMB-FL, its truncated constructs, and pGSDMD were transfected into HEK-293T cells. After 24 h, cell lysates and supernatants were collected to assess pyroptosis by LDH release assay (**D**) and PI staining (**E**). (**F–G**) pGSDMB-FL or its truncated constructs were transfected into IPI-2I cells for 24 h, followed by PEDV infection. At 24 hpi, viral replication was assessed by RT-qPCR (**F**) or IFA (**G**), respectively. Results are expressed as means ± standard deviation from three independent experiments. *P* values were analyzed using Student’s *t-*test. *P < 0.05; **P < 0.01; ***P < 0.001; ****P < 0.0001; ns, not significant.

Previous study has demonstrated that hGSDMB can be cleaved by hCaspase-1, releasing its NTD and subsequently inducing pyroptosis in human embryonic kidney 293T (HEK-293T) cells (31). Sequence alignment revealed that the amino acids at the putative cleavage site are conserved between hGSDMB and pGSDMB (Fig. 4A), suggesting a similar activation mechanism. We therefore hypothesized that the N-terminal fragment of pGSDMB (pGSDMB_1–237_) could induce pyroptosis and thereby restrict PEDV replication. To test this hypothesis, as shown in Fig. 4B, we generated two truncated mutants of pGSDMB: the NTD (pGSDMB_1–237_) and the CTD (pGSDMB_238–413_). Simultaneously, the three-dimensional structure of pGSDMB was predicted using the AlphaFold3 online platform, with the NTD highlighted in blue and the CTD highlighted in red (Fig. 4C). To investigate the pyroptotic potential of pGSDMB_1–237_, HEK-293T cells were transfected with plasmids encoding pGSDMB-FL, pGSDMB_1–237_, pGSDMB_238–413_, or pGSDMD_1–279_, with the latter serving as a positive control (22). Overexpression of the pGSDMB_1–237_ led to a significant increase in LDH release and PI^+^ cell percentages, whereas neither pGSDMB-FL nor the pGSDMB_238–413_ induced such effects (Fig. 4D and E), indicating that amino acids (aa) 1–237 constitute the critical region responsible for pGSDMB-mediated pyroptosis. To investigate the relationship between pGSDMB-induced pyroptosis and PEDV replication, we transfected IPI-2I cells with pGSDMB-FL or its truncated mutants, followed by PEDV infection. As expected, the pyroptosis-competent pGSDMB_1–237_ markedly inhibited PEDV replication, whereas the pGSDMB_238–413_ had no such effect, which was consistent with the IFA results (Fig. 4F and G). Collectively, the NTD (1–237 aa) of pGSDMB mediates pyroptosis and suppresses PEDV replication.

### PEDV infection triggers D237 cleavage of pGSDMB, releasing pGSDMB_1–237_ to mediate pyroptosis

**Fig 5:**
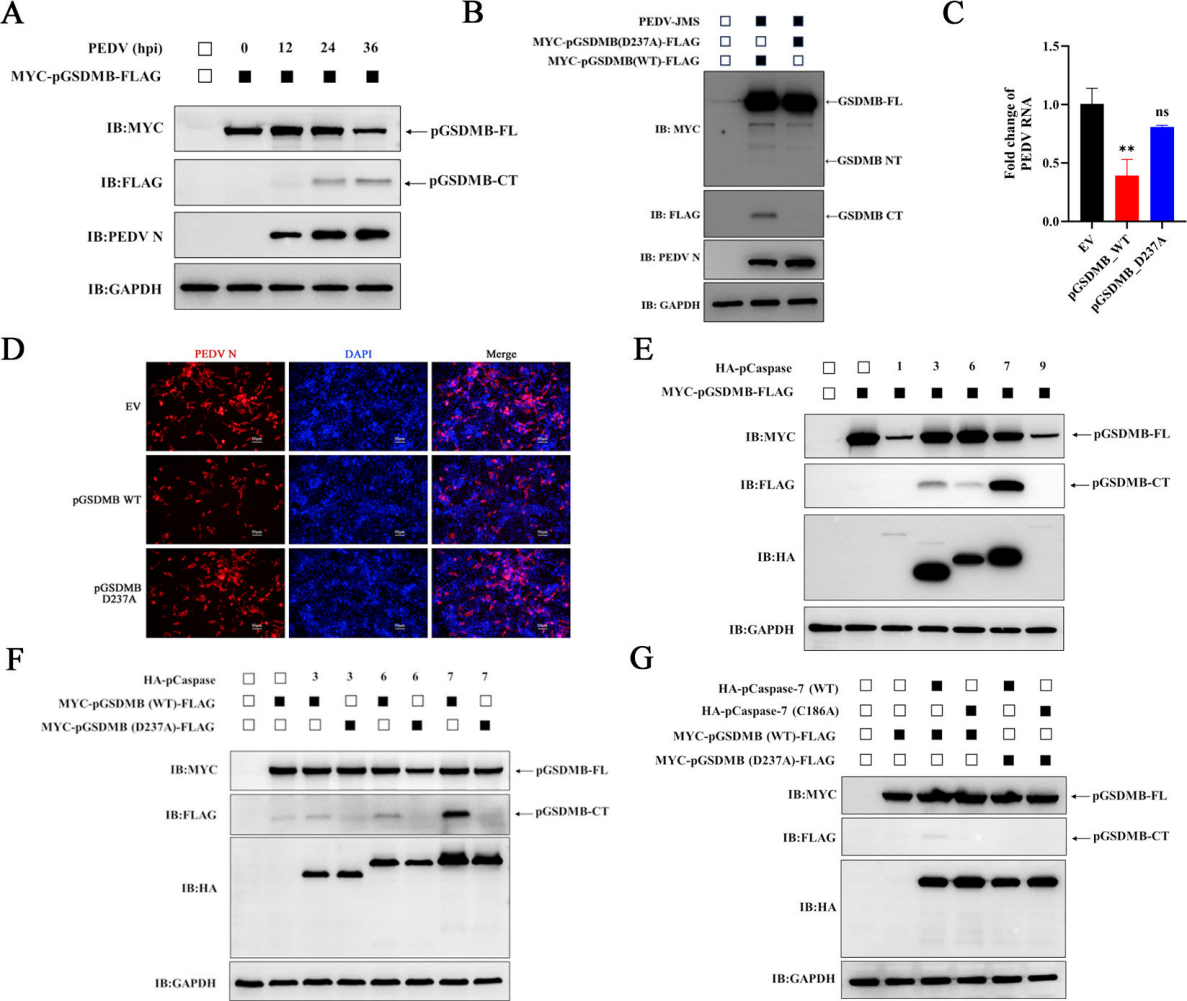
PEDV infection triggers D237 cleavage of pGSDMB, releasing pGSDMB_1–237_ to mediate pyroptosis. (**A**) Vero-E6 cells were transfected with pGSDMB for 24 h prior to PEDV infection. Samples were collected at specified time points post-infection, and pGSDMB cleavage was analyzed by western blotting. (**B–D**) Vero-E6 cells were transfected with wild-type pGSDMB or the D237A mutant for 24 h, followed by PEDV infection. At 24 hpi, pGSDMB cleavage was analyzed by western blotting (**B**); viral replication was assessed by RT-qPCR (**C**) or IFA (**D**). (**E**) HEK-293T cells were co-transfected with pGSDMB and different pCaspases (1, 3, 6, 7, or 9). After 24 h, pGSDMB cleavage was analyzed by western blotting. (**F**) Vero-E6 cells were co-transfected with wild-type pGSDMB or the D237A mutant along with pCaspase-3, pCaspase-6, or pCaspase-7. After 24 h, pGSDMB cleavage was analyzed by western blotting. (**G**) HEK-293T cells were co-transfected with wild-type or D237A mutant pGSDMB along with pCaspase-7 or its C186A mutant. After 24 h, pGSDMB cleavage was analyzed by western blotting. Results are expressed as means ± standard deviation from three independent experiments. *P* values were analyzed using Student’s *t-*test. *P < 0.05; **P < 0.01; ***P < 0.001; ****P < 0.0001; ns, not significant.

Building on the finding that pGSDMB_1–237_ induces pyroptosis and restricts PEDV replication (Fig. 4), we asked whether PEDV infection generates this functional fragment. To address this, we transfected IPI-2I cells with pGSDMB for 24 h, infected them with PEDV, and analyzed pGSDMB cleavage at the indicated time points by western blotting. As shown in Fig. 5A, the expression of full-length pGSDMB (pGSDMB-FL) gradually decreased, while a C-terminal cleavage fragment (pGSDMB-CT) of approximately 19 kDa progressively accumulated starting from 12 hpi, coinciding with the time point when pGSDMB suppresses PEDV replication (Fig. 1D). To determine whether pGSDMB-CT is generated through cleavage at D237, we constructed a pGSDMB mutant in which the aspartic acid (D) at the putative cleavage site was replaced with alanine (A) and conducted similar experiments. Cells expressing the pGSDMB (D237A) mutant failed to generate either pGSDMB-NT (~27 kDa) or pGSDMB-CT (~19 kDa) fragments upon PEDV infection (Fig. 5B). Simultaneously, we assessed the effect of the D237A mutation on pGSDMB’s ability to suppress PEDV replication. Both RT-qPCR and IFA consistently showed that the mutant lost its inhibitory effect on PEDV replication (Fig. 5C and D). These results indicate that PEDV infection induces cleavage of pGSDMB at D237, generating the pGSDMB_1–237_ fragment, which possesses both pyroptotic activity and antiviral function.

Activation of GSDMs typically requires cleavage by specific proteases, such as caspases, after which the released NTDs oligomerize and form pores in the cell membrane, ultimately leading to pyroptosis (32). To investigate whether pGSDMB-induced pyroptosis is mediated by caspases, we co-transfected HEK-293T cells with pGSDMB and various caspases, followed by western blotting analysis to assess pGSDMB cleavage. Interestingly, pGSDMB was cleaved by pCaspase-3, pCaspase-6, and pCaspase-7 to generate the pGSDMB-CT fragment, whereas pCaspase-1 and pCaspase-9 showed no such activity (Fig. 5E). As expected, mutation of the D237 residue abolished the ability of pCaspase-3, pCaspase-6, and pCaspase-7 to cleave pGSDMB, indicating that D237 is the critical site for caspase-mediated cleavage of pGSDMB (Fig. 5F), consistent with the phenomenon observed during PEDV infection (Fig. 5B). Given that pCaspase-7 exhibited the most prominent cleavage of pGSDMB, we further investigated whether this effect depends on its enzymatic activity. To this end, we co-transfected cells with pGSDMB and either wild-type pCaspase-7 or its catalytically inactive mutant (C186A) and examined the cleavage of pGSDMB by western blotting. As shown in Fig. 5G, pCaspase-7 cleaved pGSDMB to generate the pGSDMB-CT fragment, whereas the catalytically inactive mutant pCaspase-7 (C186A) completely lost this ability, indicating that pCaspase-7-mediated cleavage of pGSDMB at D237 is dependent on its enzymatic activity. Together, these results demonstrate that PEDV infection triggers cleavage of pGSDMB at D237, releasing the N-terminal fragment (pGSDMB_1–237_) responsible for pyroptosis, thereby restricting viral replication, a process mediated by pCaspase-3, pCaspase-6, and pCaspase-7.

### PEDV nsp1 and nsp15 suppress pGSDMB expression

**Fig 6:**
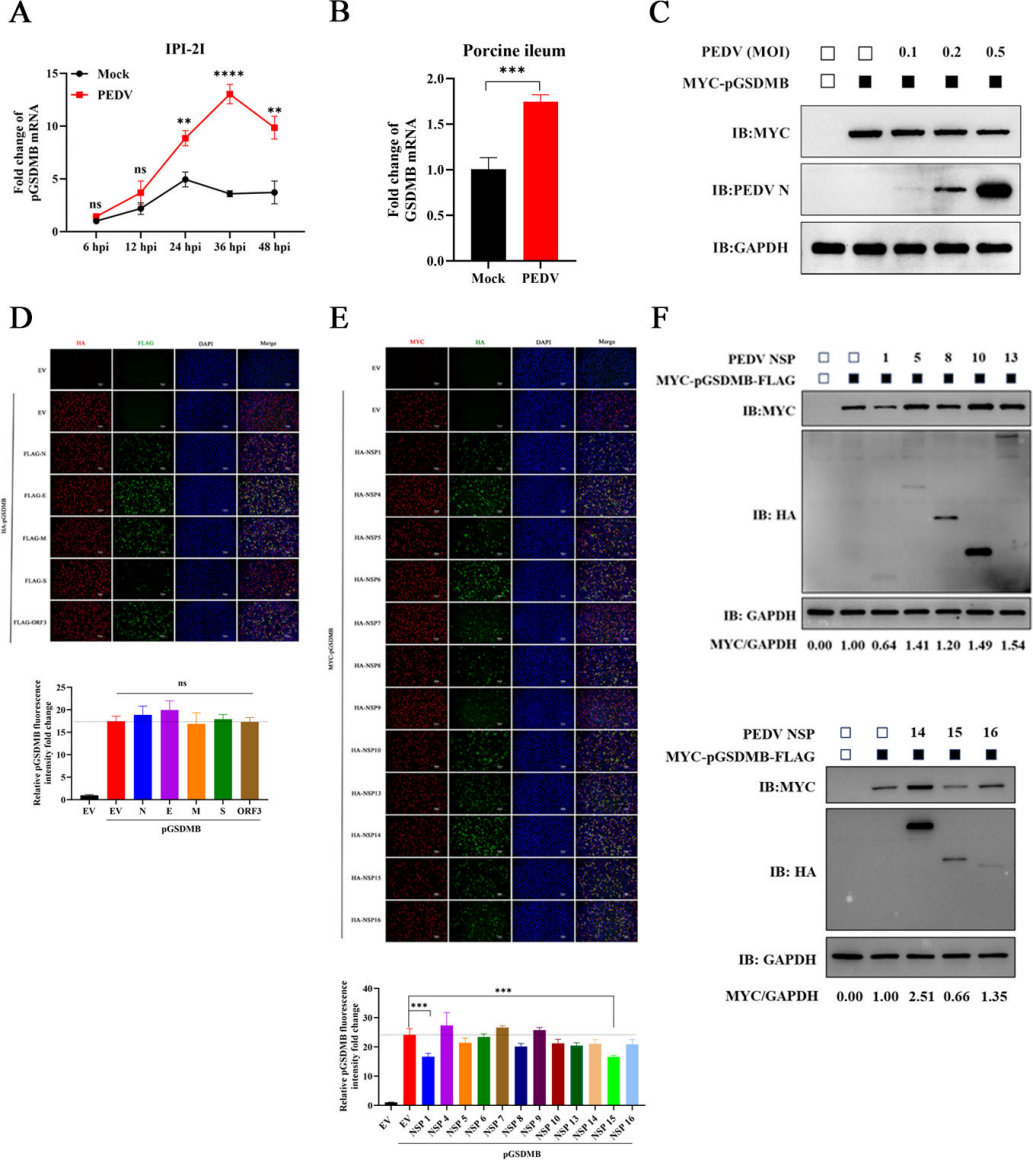
PEDV nsp1 and nsp15 suppress pGSDMB expression. (**A**) Following PEDV infection of IPI-2I cells, pGSDMB mRNA expression was measured at the indicated time points by RT-qPCR. (**B**) RT-qPCR analysis of pGSDMB mRNA expression in the ileum of piglets following PEDV infection. (**C**) Vero-E6 cells were transfected with pGSDMB for 24 h and then infected with PEDV at different MOIs. At 6 hpi, pGSDMB protein expression was analyzed by western blotting. (**D–E**) Vero-E6 cells were transfected with PEDV structural proteins (**D**) or nonstructural proteins (**E**) for 24 h, and pGSDMB expression was analyzed by IFA. (**F**) Western blotting analysis of pGSDMB protein expression in Vero-E6 cells transfected with various PEDV nonstructural proteins. Results are expressed as means ± standard deviation from three independent experiments. *P* values were analyzed using Student’s *t-*test. *P < 0.05; **P < 0.01; ***P < 0.001; ****P < 0.0001; ns, not significant.

Given that coronaviruses develop multiple strategies to evade the host innate immunity, we next sought to determine whether PEDV modulates pGSDMB expression in turn. To this end, we examined the expression levels of pGSDMB mRNA in PEDV-infected cells using RT-qPCR. Interestingly, PEDV significantly upregulated pGSDMB transcription in IPI-2I cells starting at 24 hpi (Fig. 6A). Consistently, *in vivo* experiments confirmed that PEDV infection upregulated pGSDMB mRNA in ileal tissues (Fig. 6B). However, at 6 hpi, when pGSDMB failed to inhibit PEDV replication (Fig. 1D), PEDV markedly suppressed pGSDMB protein expression in a dose-dependent manner, indicating that PEDV may attenuate the antiviral function of pGSDMB by downregulating its protein expression (Fig. 6C). Previous study has shown that viral proteins facilitate viral immune evasion by suppressing the translation of host antiviral factors (24). Based on this, we co-transfected Vero-E6 cells with pGSDMB and various structural or nonstructural proteins of PEDV to identify viral proteins involved in regulating pGSDMB expression. IFA results revealed that PEDV nsp1 and nsp15 markedly suppressed pGSDMB protein expression, whereas no such effect was observed for other nonstructural or structural proteins (Fig. 6D and E). Similarly, western blotting further confirmed that PEDV nsp1 and nsp15 significantly suppressed pGSDMB protein expression (Fig. 6F). Thus, these results indicate that PEDV suppresses pGSDMB protein expression through nsp1 and nsp15, thereby attenuating the antiviral activity of pGSDMB.

### PEDV nsp1 (86–110 aa) and nsp15 (H226/H241) are critical for suppressing pGSDMB expression

**Fig 7:**
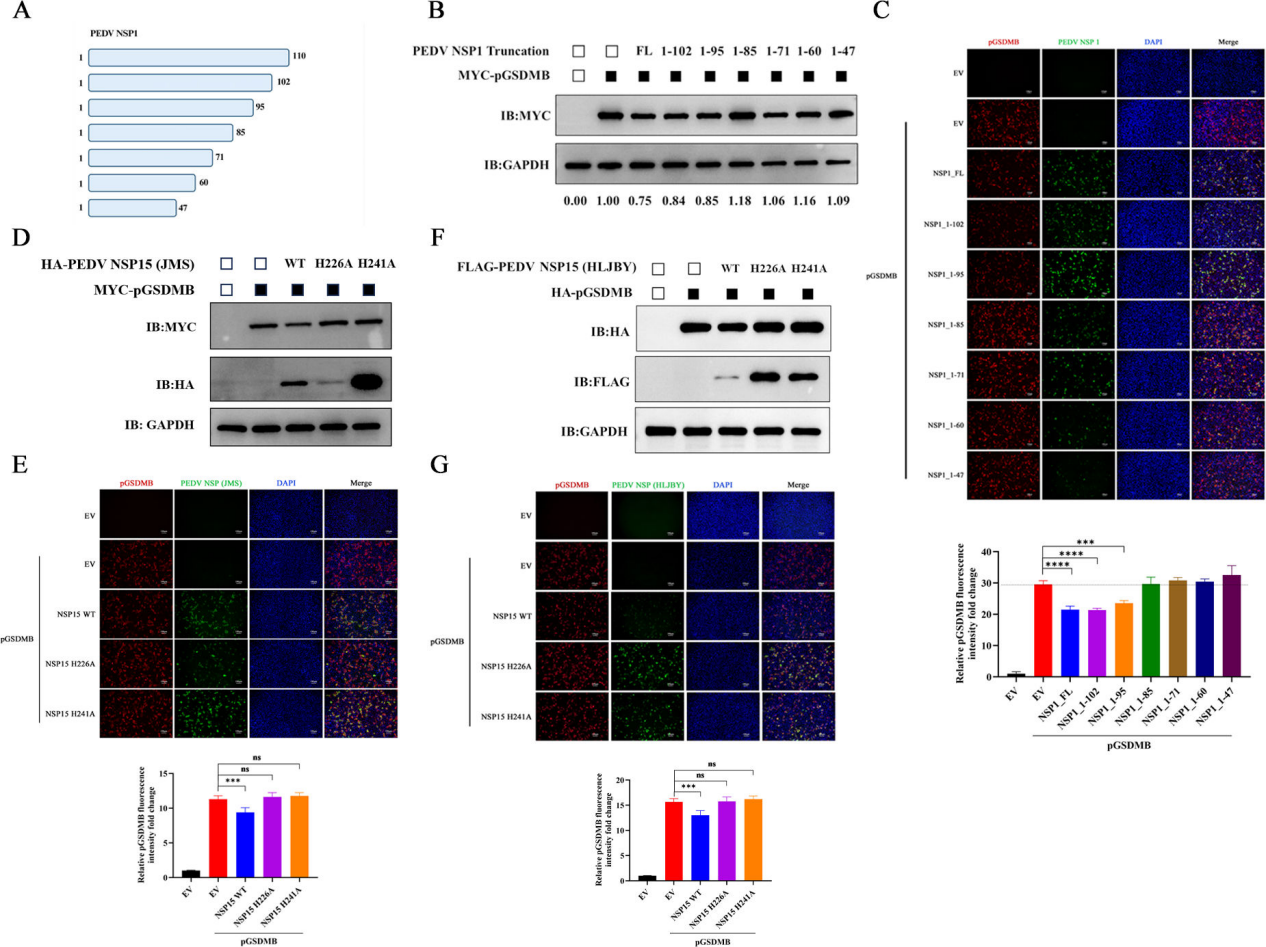
PEDV nsp1 (86–110 aa) and nsp15 (H226/H241) are critical for suppressing pGSDMB expression. (**A**) Schematic representation of full-length PEDV nsp1 and its truncated constructs. (**B–C**) Vero-E6 cells were transfected with full-length PEDV nsp1 or its truncated mutants for 24 h, and pGSDMB expression was analyzed by western blotting (**B**) or IFA (**C**), respectively. (**D–E**) Vero-E6 cells were transfected with full-length PEDV-JMS nsp15 or its catalytic mutant (nsp15 H226A and nsp15 H241A) for 24 h, and pGSDMB expression was analyzed by western blotting (**D**) or IFA (**E**). (**F–G**) Vero-E6 cells were transfected with full-length PEDV-HLJBY nsp15 or its catalytic mutant (nsp15 H226A and nsp15 H241A) for 24 h, and pGSDMB expression was analyzed by western blotting (**F**) or IFA (**G**). Results are expressed as means ± standard deviation from three independent experiments. *P* values were analyzed using Student’s *t-*test. *P < 0.05; **P < 0.01; ***P < 0.001; ****P < 0.0001; ns, not significant.

To identify the critical region of PEDV nsp1 responsible for suppressing pGSDMB expression, we constructed a series of nsp1 truncation mutants (Fig. 7A) and co-transfected them with pGSDMB into Vero-E6 cells. Both western blotting and IFA results confirmed that nsp1 (1–102 aa) and nsp1 (1–95 aa) retained the ability to suppress pGSDMB expression, whereas nsp1 (1–85 aa) and other truncation mutants lost this activity, indicating that residues 86–110 of PEDV nsp1 constitute the critical region for inhibiting pGSDMB expression (Fig. 7B and C). A recent study has shown that the coronavirus EndoU nsp15 suppresses host protein translation in a manner dependent on its catalytic activity (26). To investigate whether PEDV nsp15-mediated suppression of pGSDMB expression also depends on its enzymatic activity, we mutated the catalytic residues H226 and H241, which are essential for PEDV nsp15 EndoU activity, and generated the enzymatically inactive mutants nsp15 (H226A) and nsp15 (H241A). As shown in Fig. 7D and E, mutation of the catalytic core residues largely abolished nsp15’s ability to suppress pGSDMB expression, indicating the indispensable role of EndoU activity in mediating this inhibitory effect. Amino acid sequence alignment revealed that the catalytic residues H226 and H241 are highly conserved across different PEDV genotypes (G1, G2a, G2b, and G2c) (Fig. S2). To determine whether the inhibitory effect of PEDV nsp15 on pGSDMB is conserved across different strains, we performed the same experiments using nsp15 and its catalytic mutants from PEDV-HLJBY, a PEDV G2b strain. Consistent with previous reports (26), PEDV-HLJBY nsp15 also suppressed pGSDMB expression, whereas its catalytic mutants did not, indicating that the inhibitory effect of PEDV nsp15 on pGSDMB is not strain-specific (Fig. 7F and G). Collectively, these data indicate that 86–110 aa of PEDV nsp1 and the catalytic residues H226 and H241 of nsp15 are critical regions and residues responsible for suppressing pGSDMB expression.

### pGSDMB suppresses other SECoVs replication through pyroptosis

**Fig 8:**
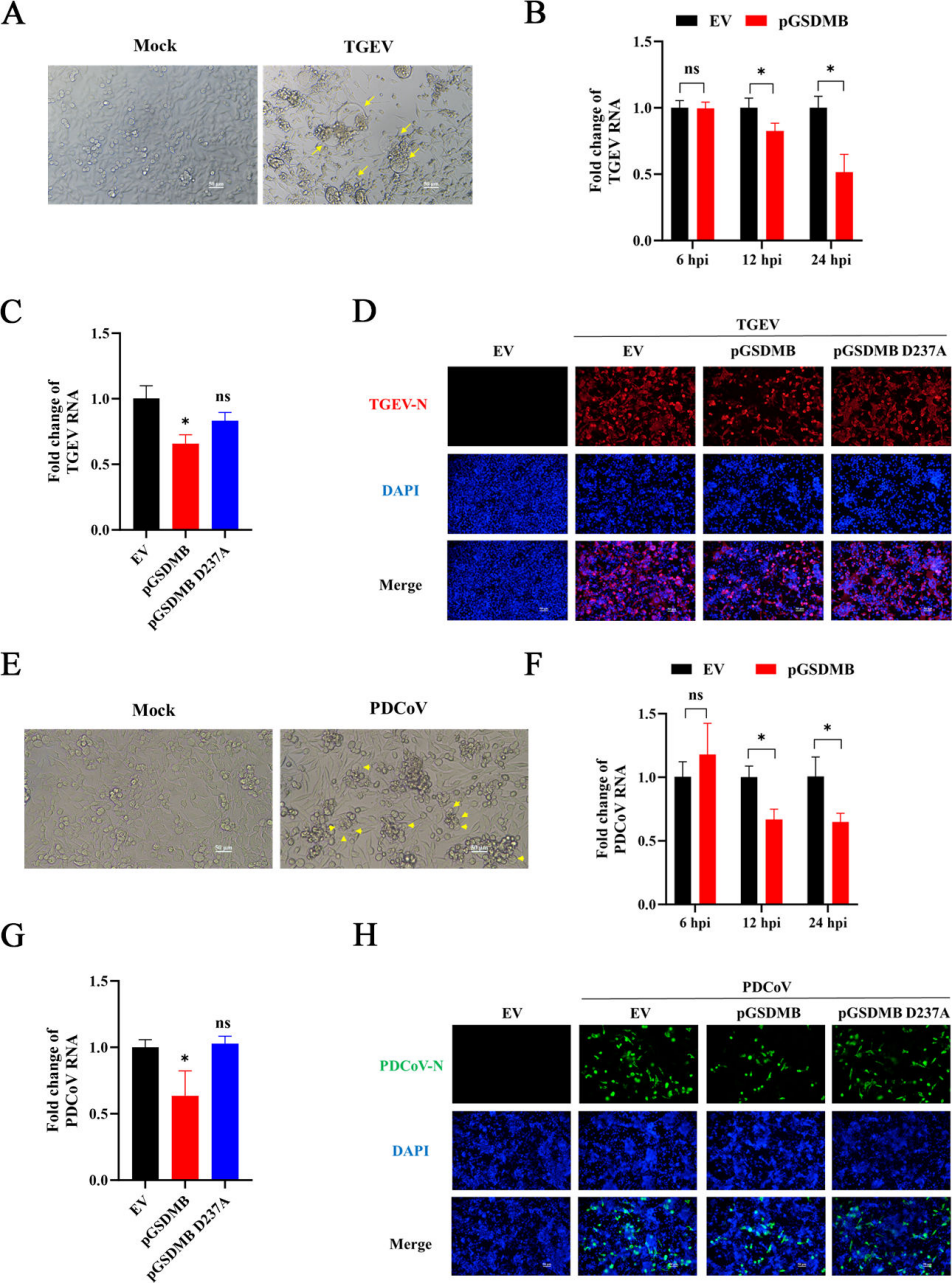
pGSDMB suppresses other SECoVs replication through pyroptosis. (**A**) Morphological changes of pyroptotic IPI-2I cells were observed under a light microscope 24 h after TGEV infection, with yellow arrows indicating pyroptotic cells. (**B**) IPI-2I cells were transfected with pGSDMB for 24 h and then infected with TGEV for the indicated times. Viral genome expression was quantified by RT-qPCR. (**C–D**) IPI-2I cells were transfected with wild-type pGSDMB or the D237A mutant for 24 h, followed by TGEV infection. Viral replication was assessed at 24 hpi by RT-qPCR (**C**) or IFA (**D**), respectively. (**E**) Pyroptotic morphological changes in IPI-2I cells were examined under a light microscope 24 h after PDCoV infection, with yellow arrows highlighting pyroptotic cells. (**F**) IPI-2I cells were transfected with pGSDMB for 24 h, followed by PDCoV infection for the indicated times, and viral genome levels were quantified by RT-qPCR. (**G–H**) IPI-2I cells were transfected with wild-type pGSDMB or the D237A mutant for 24 h, followed by PDCoV infection. At 24 hpi, viral replication was evaluated by RT-qPCR (**G**) or IFA (**H**), respectively. Results are expressed as means ± standard deviation from three independent experiments. *P* values were analyzed using Student’s *t-*test. *P < 0.05; **P < 0.01; ***P < 0.001; ****P < 0.0001; ns, not significant.

Given that pGSDMB is highly expressed in the porcine small intestine and significantly suppresses PEDV replication, we sought to determine whether the antiviral function of pGSDMB is broadly conserved across SECoVs. To this end, we selected TGEV and PDCoV as representative strains for investigation, which belong to the *Alphacoronavirus* genus and the *Deltacoronavirus* genus, respectively. Similarly, characteristic pyroptotic morphological changes were observed in TGEV-infected IPI-2I cells (Fig. 8A), and both the percentage of PI^+^ cells and LDH release increased over time (Fig. S3A and B), suggesting that TGEV infection induces pyroptosis. To assess the effect of pGSDMB on TGEV infection, IPI-2I cells were transfected with pGSDMB for 24 h prior to TGEV infection. RT-qPCR results showed that pGSDMB inhibited TGEV replication starting from 12 hpi (Fig. 8B), suggesting it exerts antiviral activity during the late stage of TGEV infection. Notably, the pGSDMB D237A mutant lost this inhibitory effect, indicating that pGSDMB restricts TGEV replication via its pyroptotic function (Fig. 8C), which was further confirmed by IFA (Fig. 8D). Consistently, a similar pattern was observed during PDCoV infection, in which PDCoV infection induced pyroptosis (Fig. 8E; Fig. S3C and D) and pGSDMB markedly suppressed viral replication, while pGSDMB D237A mutant failed to exert such an effect (Fig. 8F through H). Taken together, these data demonstrate that pGSDMB exerts broad antiviral activity against multiple SECoVs through its ability to induce pyroptosis.

## DISCUSSION

**Fig 9:**
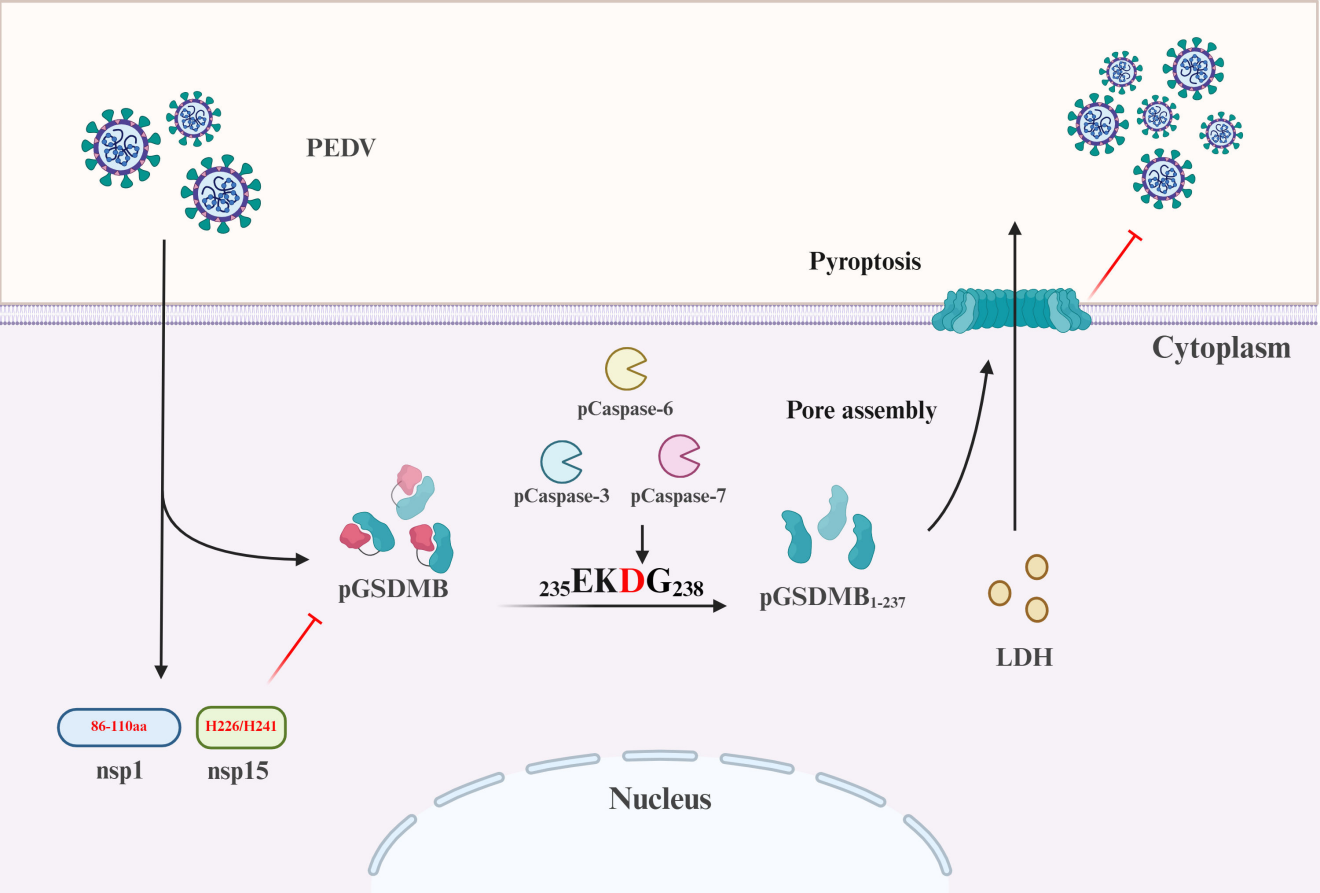
Schematic model of pGSDMB-mediated inhibition of PEDV replication through pyroptosis. PEDV infection triggers cleavage of pGSDMB at D237, releasing the pore-forming pGSDMB_1–237_ fragment, which induces pyroptosis and limits viral replication in a pCaspase-3-, pCaspase-6-, and pCaspase-7-dependent manner. In contrast, PEDV nsp1 and nsp15 inhibit pGSDMB-mediated antiviral activity by suppressing its protein expression. The 86–110 aa region of nsp1 and the catalytic residues H226 and H241 of nsp15 are critical for their respective functions. The model was created using BioRender (https://biorender.com).

GSDMs are pivotal executors of cell death in host defense and disease. While GSDMB is a well-established risk factor in asthma, its role in intestinal antiviral immunity has remained enigmatic. Our work demonstrates that pGSDMB is a potent antiviral effector against SECoVs, triggering pyroptosis to restrict viral replication. Crucially, we discover a novel immune evasion strategy wherein PEDV counteracts this defense through the synergistic suppression of pGSDMB by nsp1 and nsp15 (Fig. 9). These findings not only redefine GSDMB as a key guardian of the gut mucosa but also reveal a new front in the host-virus arms race, unveiling promising therapeutic targets for combating coronaviruses.

GSDMB is highly expressed in the epithelial cells lining organs and plays a critical and complex role in maintaining mucosal barrier integrity and orchestrating immune responses. Upregulation of GSDMB in airway epithelial cells or leukocytes appears to be a risk factor for childhood asthma onset as well as for disease severity and exacerbation (33). Indeed, knock-in mice ubiquitously expressing the GSDMB isoform three exhibit an asthmatic phenotype (7). These findings underscore the pivotal role of GSDMB in respiratory diseases. In stark contrast, within the gastrointestinal tract, a growing body of evidence establishes that intestinal epithelial cell-derived GSDMB exerts a crucial protective role against a range of threats, including inflammatory bowel disease, colorectal cancer, and enteric bacterial infections (19, 34–36). Consistently, we found that pGSDMB, which is highly expressed in the porcine small intestine, significantly inhibits SECoVs infection (Fig. 1 and 8), further underscoring the protective role of GSDMB in intestinal immunity, particularly against enteric viral infections. Our result solidifies the role of GSDMB as a broad-spectrum guardian of the intestinal mucosal barrier. The absence of a mouse or rat ortholog for GSDMB (3) positions the pig as a highly relevant functional surrogate. Thus, studying pGSDMB is not only valuable for porcine biology but also provides a critical and translationally pertinent model for elucidating the *in vivo* roles of GSDMB in human infections, cancers, asthma, and other immune pathologies.

The proteolytic activation of GSDMs is a fundamental event in programmed cell death, traditionally mediated by caspases and granzymes. However, our findings reveal a significant and complex divergence in the regulation of GSDMB, particularly between humans and pigs, underscoring that its cleavage by caspases is far from a uniform process. For instance, hCaspase-1, hCaspase-4, hCaspase-5, and hCaspase-11 specifically cleave GSDMD after the _272_FLTD_275_ in human (or _273_LLSD_276_ in mouse) sequence to induce pyroptosis (37). Granzyme B derived from natural killer cells can directly cleave hGSDME at the same site targeted by hCaspase-3, leading to the release of the effector N-terminal fragment and subsequent pore formation in the plasma membrane (38). However, the functional role of caspases in GSDMB-mediated cell death remains highly controversial. Panganiban et al. (31) reported that hCaspase-1 cleaves hGSDMB at the D236 residue to generate the hGSDMB-NT fragment and trigger pyroptosis. Another study also has found that hCaspase-1 mediates hGSDMB cleavage during monkeypox virus infection, generating an approximately 18 kDa hGSDMB-CT fragment, but the precise amino acid cleavage site was not specified (39). In contrast, Shi et al. (37) reported that hGSDMB and hGSDMC consistently resist cleavage by hCaspase-1/11 in HEK-293T cells. Unexpectedly, we found that pGSDMB was not cleaved by pCaspase-1 to generate an ~19 kDa fragment, but its expression was significantly suppressed by pCaspase-1 (Fig. 5E). Chao et al. (40) reported that hCaspase-3, hCaspase-6, and hCaspase-7 cleave hGSDMB at the _88_DNVD_92_ motif. Interestingly, we found that pCaspase-3, pCaspase-6, and pCaspase-7 cleave pGSDMB at both D92 and D237, whereas cleavage at D92 does not affect the regulation of PEDV replication, as mutation of this site did not alter the inhibitory activity of pGSDMB against PEDV (data not shown). This stark species-specific difference is likely rooted in the moderate sequence identity (55.3%) between hGSDMB and pGSDMB. Although they share similar overall folds, pronounced local structural variations can profoundly alter protease accessibility and recognition motifs (Fig. S4A and B). This divergence is consistent with other GSDMs; for instance, the activation of GSDMA is also mediated by different caspases across species (41). The precise functional consequences of various GSDMB cleavage and the full repertoire of proteases governing the pGSDMB pathway remain compelling subjects for future investigation.

It is not surprising that viruses have evolved multiple strategies to modify the proteins of GSDM family given that GSDM proteins play critical roles in the inflammatory responses and cellular deaths. For instance, ASFV protease pS273R cleaves pGSDMD_1–107_ at G107, generating fragments that impair the activity of pGSDMD_1–279_, thereby facilitating ASFV replication (42). Beyond proteases, our work reveals that coronaviruses have evolved alternative, nonproteolytic strategies to suppress the GSDM pathway. Specifically, we confirm that PEDV utilizes the accessory protein nsp1 to inhibit pGSDMB protein expression during early infection, with the 86–110 aa domain being critical for this function (Fig. 7B). This aligns with the established role of nsp1 in suppressing host translation by degrading mRNA and hijacking ribosomes (43), a function we previously showed targets antiviral genes like NLRC5 (44). In parallel to nsp1, we also identify the coronavirus nsp15 as a second viral factor capable of suppressing pGSDMB. This finding is consistent with recent reports that nsp15 modulates host RNA and translation machinery to inhibit protein synthesis (26). The dependence of this activity on the highly conserved catalytic residues H226 and H241 suggests a mechanism linked to its RNase function, which is essential for viral immune evasion (Fig. 7D through G). The deployment of two distinct viral proteins, nsp1 and nsp15, to achieve the same goal—suppressing pGSDMB—highlights the critical evolutionary pressure for coronaviruses to antagonize GSDMB-mediated host defense.

## MATERIALS AND METHODS

### Cell cultures and viruses

Porcine ileum intestinal epithelial (IPI-2I) cells, African green monkey kidney (Vero-E6; ATCC CRL-1586) cells, and HEK-293T (ATCC CRL-3216) cells were grown in Dulbecco’s modified Eagle’s medium (DMEM; Gibco, USA) supplemented with 10% fetal bovine serum (FBS; Gibco, USA) and 1% penicillin-streptomycin (Gibco, USA). All of the cells were cultured at 37 ˚C with 5% CO_2_.

The PEDV-JMS strain (GenBank No. PP461398.1), the TGEV-JMS strain (GenBank accession no: OR711347.1), and the PDCoV-NH strain (GenBank accession no: KU981062.1) were isolated and maintained in our lab.

### Antibodies

Rabbit anti-HA mAb (#C29F4) and rabbit anti-Flag mAb (#D6W5B) were purchased from Cell Signaling Technology (USA). Mouse anti-Flag mAb (#F1804) was obtained from Sigma-Aldrich (USA). Mouse anti-Myc mAb (#1A5A2), rabbit anti-GAPDH pAb (#10494-1-AP), coraLite488-conjugated goat anti-mouse IgG (H + L) (#SA00013-1), coraLite488-conjugated goat anti-rabbit IgG (H + L) (#SA00013-2), coraLite594-conjugated goat anti-mouse IgG (H + L) (#SA00013-3), and coraLite594-conjugated goat anti-rabbit IgG (H + L) (#SA00013-4) were purchased from Proteintech Group (USA). Horseradish peroxidase (HRP)-labeled goat anti-mouse IgG (H + L) (A0216) and HRP-labeled goat anti-rabbit IgG (H + L) (#A0208) were purchased from Beyotime (China). Rabbit anti-PEDV N mAb, mouse anti-TGEV N mAb, and rabbit anti-PDCoV N mAb were prepared by our lab.

### Cloning and construction of plasmids

According to the manufacturer’s instructions, plasmids were constructed using the ClonExpress Ultra one-step cloning kit (Vazyme, China) with specific primers listed in Table 1. The N-terminal Myc-tagged and C-terminal Flag-tagged pGSDMB (full-length, 1–237, and 238–413 aa) were cloned into pCMV vector. The N-terminal HA-tagged pGSDMB was cloned into the pCAGGS vector. Mutant plasmids of pGSDMB and PEDV-JMS nsp15 were generated using Mut Express II Fast Mutagenesis Kit V2 (Vazyme, China). The PEDV viral protein expression vectors, pCaspase-1, pCaspase-3, pCaspase-6, pCaspase-7, pCaspase-9, and pCaspase-7 (C186A) were available in our laboratory. The wild-type and mutant PEDV-HLJBY nsp15 plasmids were kindly provided by Dr. Ying Liao, Shanghai Veterinary Research Institute, Chinese Academy of Agricultural Sciences.

**TABLE 1:** Sequences of primers used for PCR^*a*^

Primer name	Sequence (5′−3′)
pGSDMB-FL-F	ACCGAGATCTCTCGAGGTACCATGCCCAAGGAGTTTGAGGC
pGSDMB-FL-R	CATGTCTGGATCCCCGCGGCCGCTTACTTGTCATCGTCGTCCTT GTAATCGGAAGACACAGAGGTAGGCTTCT
pGSDMB (1–237)-F	ACCGAGATCTCTCGAGGTACCATGCCCAAGGAGTTTGAGGC
pGSDMB (1–237)-R	CATGTCTGGATCCCCGCGGCCGCTTACTTGTCATCGTCGTCCTT GTAATCATCTTTCTCGTGTGGAAAGG
pGSDMB (238–413)-F	TTTAAACTTAAGCTTGGTACCATGGATTACAAGGACGACGATGAC AAGGGTGGTGCATCTTGCTTAG
pGSDMB (238–413)-R	AACGGGCCCTCTAGACTCGAGTTAGGAAGACACAGAGGTAGGC
pGSDMB (D237A)-F	ACGAGAAAGCTGGTGGTGCATCTTGCTTAGGG
pGSDMB (D237A)-R	ACCACCAGCTTTCTCGTGTGGAAAGGATTTTG
PEDV-JMS nsp15 (H226A)-F	CTTTGAGGCTGTTGTGTATGGTGATGTTTCAAAAAC
PEDV-JMS nsp15 (H226A)-R	ACACAACAGCCTCAAAGCCGTAATCTTCAAGTCC
PEDV-JMS nsp15 (H241A)-F	GGTGGTTTAGCTCTACTAATTTCGCAGGTGCGTCT
PEDV-JMS nsp15 (H241A)-R	AGTAGAGCTAAACCACCAAGGGTGGTTTTTGA

^*a*^ F: forward primer; R: reverse primer.

### Virus infection and transfection

IPI-2I cells and Vero-E6 cells were infected with PEDV, TGEV, or PDCoV at the indicated MOI or mock-infected with DMEM. After incubation at 37°C for 2 h, cells were washed three times with serum-free DMEM to remove unbound virus and maintained in serum-free DMEM containing 5 µg/mL of trypsin without EDTA (Gibco, USA) at 37°C until harvest.

IPI-2I cells, Vero-E6 cells, and HEK-293T cells were seeded in multi-well plates at 80% confluence and transfected with plasmids using Lipofectamine 2000 reagent (Invitrogen, USA) or Lipo8000 transfection reagent (Beyotime, China) according to the manufacturer’s instructions. After 24 h of transfection, the cells were harvested or subjected to viral infection assays.

### RNA extraction and RT-qPCR

According to the manufacturer’s instructions, total RNA was extracted using the VAMNE Magnetic Cell/Tissue Total RNA Kit (Vazyme, China) and reverse-transcribed into cDNAs using the PrimeScript II First Strand cDNA Synthesis Kit (Takara, Japan).

RT-qPCR analysis was performed on the LightCycler 480 II system (Roche, Switzerland) using 2 × Taq Pro Universal SYBR qPCR Master Mix (Vazyme, China). Gene-specific primers are listed in Table 2. Relative mRNA expression levels were calculated using the 2^^−ΔΔCT^ method and normalized to GAPDH.

**TABLE 2:** Sequences of primers used for RT-qPCR^*a*^

Primer name	Sequence (5′−3′)
pGSDMB-qPCR-F	TTGACCCCGAGGCACGAACT
pGSDMB-qPCR-R	GAAGCGGGATGGCGAGGATAG
GAPDH-qPCR-F	CCTTCCGTGTCCCTACTGCCAAC
GAPDH-qPCR-R	GACGCCTGCTTCACCACCTTCT
PEDV-qPCR-F	TGTTGCACACTTATTGGCAGGCT
PEDV-qPCR-R	ATTTGCCGTCATAATAAGCTGCT
IFN-β-qPCR-F	AGCACTGGCTGGAATGAAAC
IFN-β-qPCR-R	TCCAGGATTGTCTCCAGGTC
OASL-qPCR-F	TCCCTGGGAAGAATGTGCAG
OASL-qPCR-R	CCCTGGCAAGAGCATAGTGT
ISG15-qPCR-F	AGCATGGTCCTGTTGATGGTG
ISG15-qPCR-R	CAGAAATGGTCAGCTTGCACG
IFN-λ1-qPCR-F	CCACGTCGAACTTCAGGCTT
IFN-λ1-qPCR-R	ATGTGCAAGTCTCCACTGGT

^*a*^ F: forward primer; R: reverse primer.

### Immunofluorescence assay

IFA was performed as described previously (45). Briefly, cells subjected to various experimental treatments were fixed with pre-cold 4% paraformaldehyde at 4°C for 30 min and washed three times with phosphate-buffered saline (PBS). The cells were then permeabilized with 0.2% Triton X-100 at room temperature for 30 min and blocked with PBS containing 5% FBS and 5% skim milk (Sigma-Aldrich, USA) at 37°C for 1 h. Subsequently, cells were incubated with the appropriate primary antibodies at 37°C for 2 h, followed by incubation with corallite 488- or 594-conjugated secondary antibodies at 37°C for 2 h. After three additional PBS washes, nuclei were stained with DAPI (4′,6-diamidino-2-phenylindole). Fluorescence images were acquired using a Nikon Ti2-E fluorescence microscope. The fluorescence intensity was quantified using ImageJ (version 1.54d).

### Western blotting

Cells seeded in six-well plates were lysed on ice with RIPA lysis buffer (Beyotime, China) supplemented with 1% phenylmethylsulfonyl fluoride (Beyotime, China). Equal amounts of protein were separated by SDS-PAGE and transferred onto polyvinylidene fluoride (PVDF) membranes (Millipore, USA). Membranes were blocked with 5% skim milk in Tris-­buffered saline with Tween-20 (TBST; Solarbio, China) for 1 h at room temperature, followed by incubation with the corresponding primary antibodies overnight at 4°C. After three washes with TBST, membranes were incubated with appropriate HRP-conjugated secondary antibodies for 1 h at room temperature. Antibody binding was detected using a standard ECL detection kit (Beyotime, China) and visualized with a Tanon 4800 Chemiluminescent Imaging System (Bio Tanon, China). GAPDH was used as a loading control. The band intensities in the western blotting were quantified using ImageJ (version 1.54d).

### Cell cytotoxicity and PI staining

Cells were seeded in 48-well plates and subjected to experimental treatments. Following treatment, culture supernatants were collected for LDH release measurement using the CytoTox 96 Non-Radioactive Cytotoxicity Assay Kit (Promega, USA), and cells were stained with PI (Sigma-Aldrich, USA) for 20 min at 37°C for viability assessment.

### Generation of pGSDMB-KO cell line

Based on our previous report (22), a pGSDMB-KO cell line was generated using CRISPR/Cas9 technology. Briefly, a single guide RNA (sgRNA) targeting the exon 2 of pGSDMB (listed in Table 3) was designed using the online tool CHOPCHOP (https://chopchop.cbu.uib.no/) and cloned into the pX459 vector, which was subsequently transfected into IPI-2I cells. Single-cell clones were obtained by puromycin selection followed by fluorescence-activated cell sorting, and complete knockout of pGSDMB was confirmed by Sanger sequencing.

**TABLE 3:** Sequences of primers used for pGSDMB-KO cell line^*a*^

Primer name	Sequence (5′−3′)
sgRNA-pGSDMB-F	CACCGCACAATGCCCAAGGAGTTTG
sgRNA-pGSDMB-R	AAACCAAACTCCTTGGGCATTGTGC
pGSDMB-KO-F	TAGGGAACACCACACCCTCC
pGSDMB-KO-R	AGTCGGGTTGGGTAGGAGTT

^*a*^ F: forward primer; R: reverse primer.

### Viral titration assay

The median tissue culture infectious dose (TCID_₅₀_) assay was performed as previously described to determine viral titers (46). Briefly, cells were seeded into 96-well plates and cultured overnight until reaching full confluence. Serial 10-fold dilutions of the indicated viral supernatants (10^−1^ to 10^−8^) were prepared and added to the cells, with eight replicate wells per dilution. After 3–4 days, cytopathic effects were examined and recorded using an inverted microscope. Viral titers were calculated using the Reed-Muench method and expressed as TCID_₅₀_.

### Experimental infection of piglets

As previously described (44), 5-day-old specific-pathogen-free (SPF) piglets confirmed negative for PDCoV, porcine rotavirus, PEDV, and TGEV were randomly assigned to two groups (*n* = 3 per group). Piglets in the experimental group were orally inoculated with 1 mL of PEDV suspension containing 1 × 10³ TCID_₅₀_, whereas control piglets received 1 mL of DMEM. All animals were euthanized at 72-h post-inoculation, and tissue samples were subsequently collected for analysis.

### Amino acid sequence alignment and protein structure prediction

Amino acid sequences of porcine GSDMB (GenBank: XP_013835150.2) and hGSDMB (GenBank: KAI4049234.1) were downloaded from the NCBI database. Multiple sequence alignments were performed using MEGA12, and secondary structure elements were annotated with ESPript 3.0 (https://espript.ibcp.fr/ESPript/ESPript/). The tertiary structures of the proteins were predicted using SWISS-MODEL (https://swissmodel.expasy.org/interactive) and AlphaFold3 (https://alphafoldserver.com/).

### Statistical analysis

All experiments were performed with a minimum of three independent biological replicates, each conducted in triplicate for technical repeats. Data were analyzed using GraphPad Prism 8.0.2 (GraphPad Software, USA). Statistical significance was determined using Student’s *t*-test, with thresholds defined as follows: *P* < 0.05 (*), *P* < 0.01 (**), *P* < 0.001 (***), *P* < 0.0001 (****), and ns (not significant).

## Data Availability

All data are available in the main text or the supplemental material.

## SUPPLEMENTAL MATERIAL

Fig. S1 mbio.02904-25-s0001.tif Identification of pGSDMB-KO cell lines.

Fig. S2 mbio.02904-25-s0002.tif Amino acid sequence alignment of nsp15 from different PEDV strains.

Fig. S3 mbio.02904-25-s0003.tif TGEV and PDCoV infection induced pyroptosis.

Fig. S4 mbio.02904-25-s0004.tif Comparative analysis of the secondary and tertiary structures of hGSDMB and pGSDMB.

Legends mbio.02904-25-s0005.docx Supplemental figure legends.

ASM does not own the copyrights to Supplemental Material that may be linked to, or accessed through, an article. The authors have granted ASM a non-exclusive, world-wide license to publish the Supplemental Material files. Please contact the corresponding author directly for reuse.
