# Supplementary figures and images for: Gasdermin B-mediated pyroptosis as a host defense against swine enteric coronaviruses and its antagonism by PEDV
Source: mBio. 2025 Dec 18;17(2):e02904-25. doi: 10.1128/mbio.02904-25 (PMC12892955; doi:10.1128/mbio.02904-25)

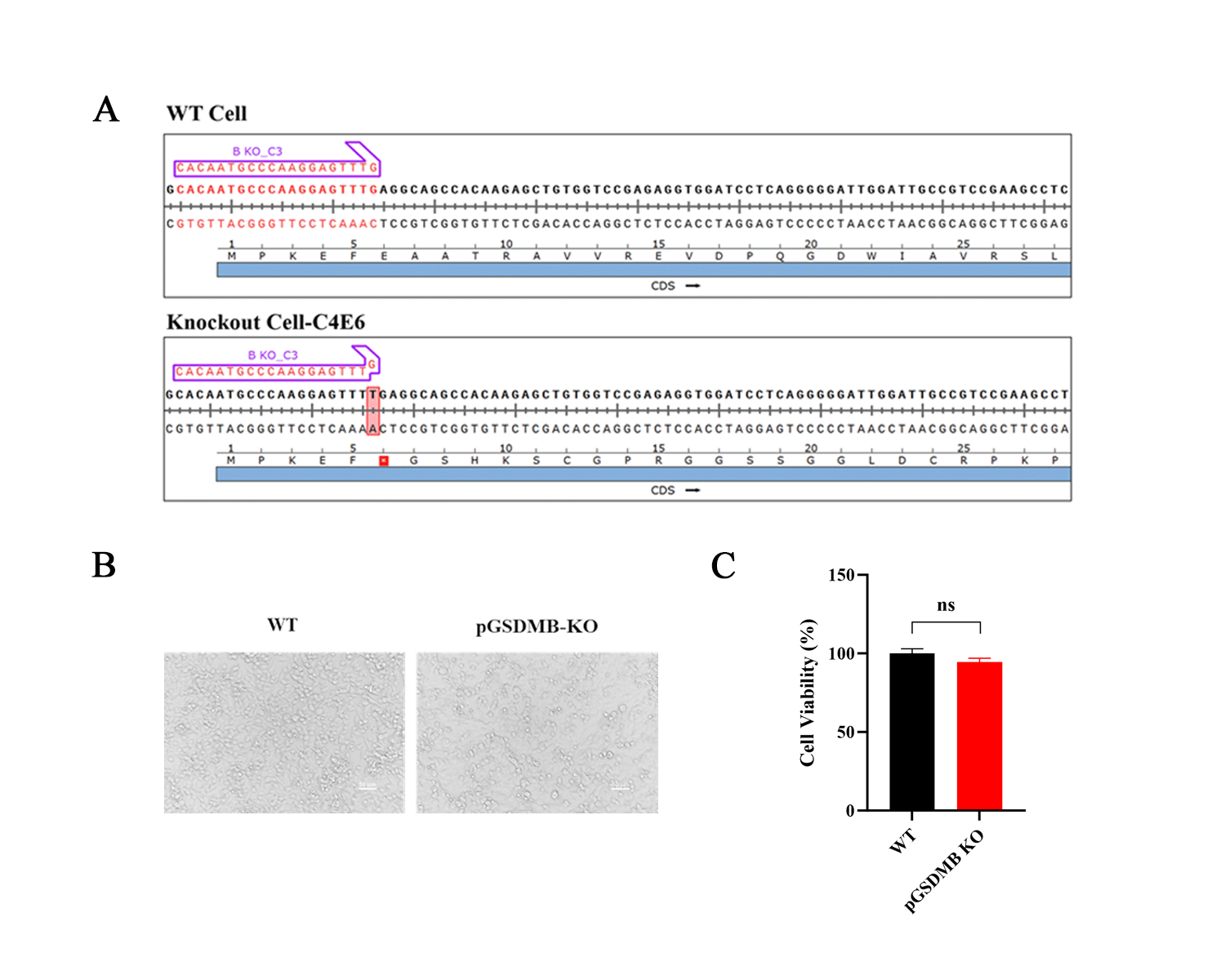

Supplement: Fig. S1 — Identification of pGSDMB-KO cell lines. [file mbio.02904-25-s0001.tif]

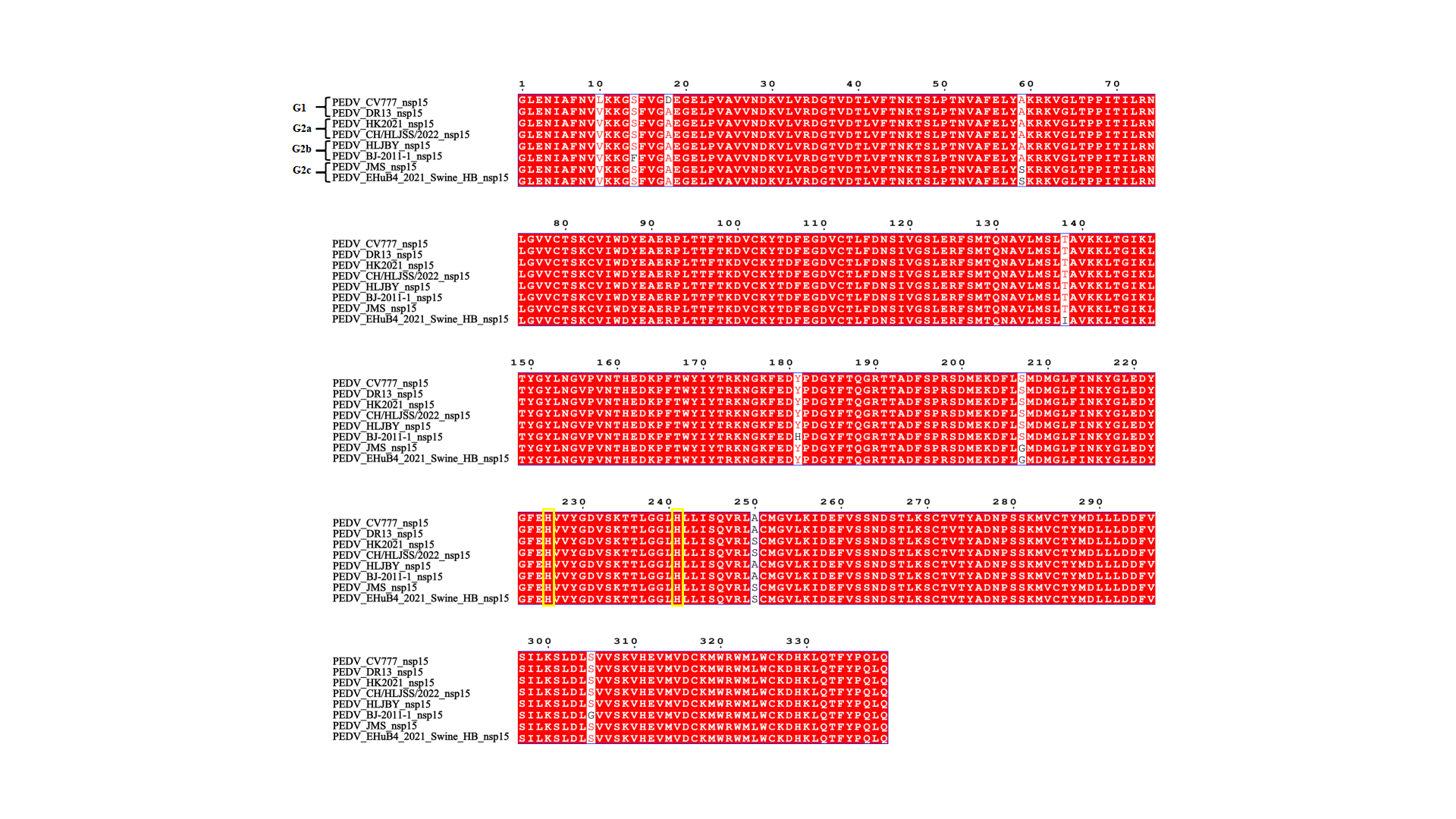

Supplement: Fig. S2 — Amino acid sequence alignment of nsp15 from different PEDV strains. [file mbio.02904-25-s0002.tif]

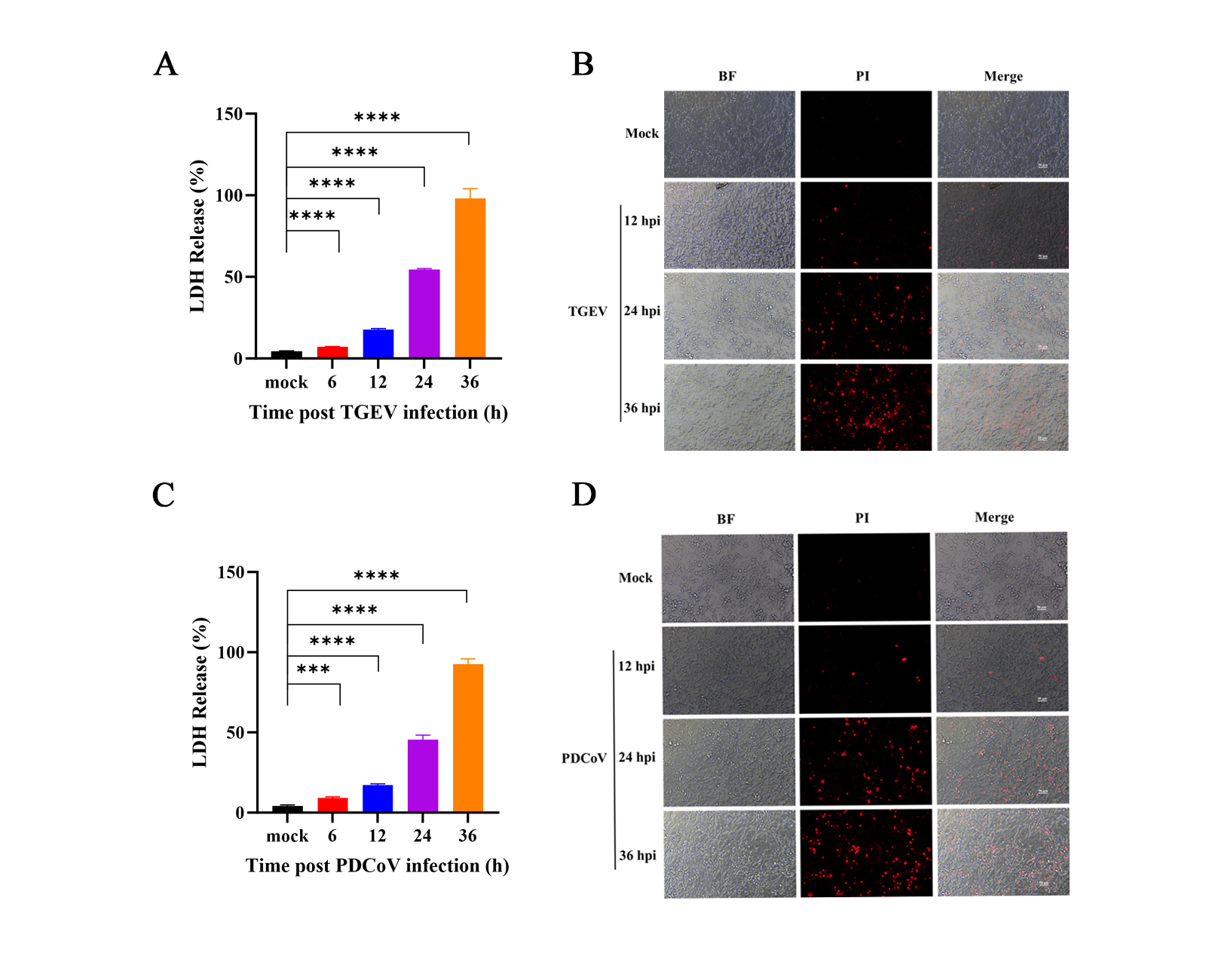

Supplement: Fig. S3 — TGEV and PDCoV infection induced pyroptosis. [file mbio.02904-25-s0003.tif]

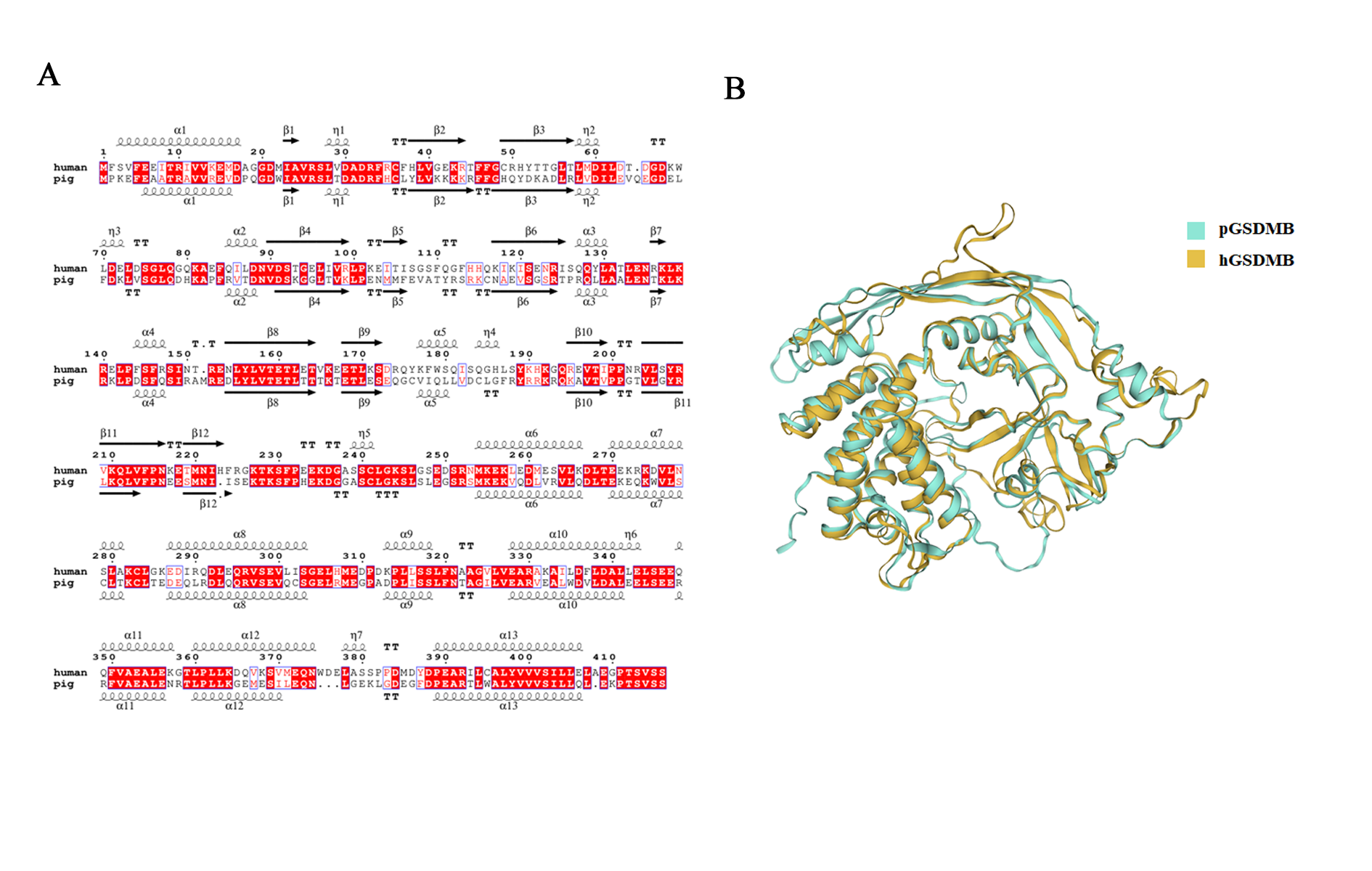

Supplement: Fig. S4 — Comparative analysis of the secondary and tertiary structures of hGSDMB and pGSDMB. [file mbio.02904-25-s0004.tif]
