# Supplementary material for: Gasdermin B-mediated pyroptosis as a host defense against swine enteric coronaviruses and its antagonism by PEDV
Source: mBio. 2025 Dec 18;17(2):e02904-25. doi: 10.1128/mbio.02904-25 (PMC12892955; doi:10.1128/mbio.02904-25)
Supplement: Legends — Supplemental figure legends. [file mbio.02904-25-s0005.docx]

**Supplementary Figure legends**

**Supplementary Fig. 1.** **Identification of pGSDMB KO cell lines.**

**(A)** Identification of pGSDMB KO cell lines by Sanger sequencing. pGSDMB KO cell line (C4E6) harbors a T insertion after the 15th base of pGSDMB, resulting in a frameshift mutation that converts the sixth codon into a stop codon, thereby causing premature termination of protein translation. **(B)** The proliferation and morphology of wild-type (WT) and pGSDMB KO IPI-2I cells were assessed using light microscopy. **(C)** The cell viability of wild-type and pGSDMB KO cell lines was determined using the CellTiter-Glo Luminescent Cell Viability Assay. Results are expressed as means ± standard deviation from three independent experiments. *P* values were analyzed using Student’s *t-*test. **P <* 0.05; ***P <* 0.01; ****P <* 0.001; *****P <* 0.0001; ns, not significant.

**Supplementary Fig. 2. Amino acid sequence alignment of nsp15 from different PEDV strains.**

Amino acid sequences of nsp15 from different PEDV strains were retrieved from NCBI database. Sequence alignments were performed using MEGA12, and the secondary structure elements were annotated with ESPript 3.0.

**Supplementary Fig. 3. TGEV and PDCoV infection induced** **pyroptosis.**

**(A-B)** Following TGEV infection of IPI-2I cells, pyroptosis was assessed at different time points by measuring LDH release in the supernatant (A) or by PI staining (B), with red fluorescence indicating PI⁺ cells. **(C-D)** Pyroptosis in PDCoV-infected IPI-2I cells was evaluated at different time points by LDH release assays of culture supernatants (C) and by PI staining (D). Results are expressed as means ± standard deviation from three independent experiments. *P* values were analyzed using Student’s *t-*test. **P <* 0.05; ***P <* 0.01; ****P <* 0.001; *****P <* 0.0001; ns, not significant.

**Supplementary Fig. 4. Comparative analysis of the secondary and tertiary structures of hGSDMB and pGSDMB.**

**(A)** Amino acid sequences of porcine GSDMB (Genbank: XP_013835150. 2) and human GSDMB (Genbank: KAI4049234. 1) were downloaded from the NCBI database. Multiple sequence alignments were performed using MEGA12, and secondary structure elements were annotated with ESPript 3.0. **(B)** The tertiary structures of hGSDMB (yellow) and pGSDMB (blue) were predicted using SWISS-MODEL.
